# Trem2 promotes foamy macrophage lipid uptake and survival in atherosclerosis

**DOI:** 10.1038/s44161-023-00354-3

**Published:** 2023-10-30

**Authors:** Michael T. Patterson, Maria M. Firulyova, Yingzheng Xu, Hannah Hillman, Courtney Bishop, Alisha Zhu, Grant H. Hickok, Patricia R. Schrank, Christine E. Ronayne, Zakariya Caillot, Gavin Fredrickson, Ainsley E. Kennedy, Nisha Acharya, Jaap G. Neels, Giulia Chinetti, Xavier Revelo, Ingunn M. Stromnes, Stoyan Ivanov, Tyler D. Bold, Konstantin Zaitsev, Jesse W. Williams

**Affiliations:** 1 Center for Immunology, University of Minnesota, Minneapolis, MN USA; 2 Department of Integrative Biology and Physiology, University of Minnesota, Minneapolis, MN USA; 3 ITMO University, Saint Petersburg, Russia; 4 Almazov National Medical Research Centre, Saint Petersburg, Russia; 5 Department of Medicine, University of Minnesota, Minneapolis, MN USA; 6 Université Côte d’Azur, CNRS, LP2M, Nice, France; 7 Université Côte d’Azur, INSERM, C3M, Nice, France; 8 Université Côte d’Azur, CHU, INSERM, C3M, Nice, France; 9 Department of Microbiology and Immunology, University of Minnesota, Minneapolis, MN USA

**Keywords:** Atherosclerosis, Foam cells

## Abstract

Atherosclerosis is driven by the expansion of cholesterol-loaded ‘foamy’ macrophages in the arterial intima. Factors regulating foamy macrophage differentiation and survival in plaque remain poorly understood. Here we show, using trajectory analysis of integrated single-cell RNA sequencing data and a genome-wide CRISPR screen, that triggering receptor expressed on myeloid cells 2 (Trem2) is associated with foamy macrophage specification. Loss of Trem2 led to a reduced ability of foamy macrophages to take up oxidized low-density lipoprotein (oxLDL). Myeloid-specific deletion of Trem2 showed an attenuation of plaque progression, even when targeted in established atherosclerotic lesions, and was independent of changes in circulating cytokines, monocyte recruitment or cholesterol levels. Mechanistically, we link Trem2-deficient macrophages with a failure to upregulate cholesterol efflux molecules, resulting in impaired proliferation and survival. Overall, we identify Trem2 as a regulator of foamy macrophage differentiation and atherosclerotic plaque growth and as a putative therapeutic target for atherosclerosis.

## Abstract (web summary)

Patterson, Firulyova, et al. report that TREM2 is a key regulator of foamy macrophage differentiation. Myeloid-specific deletion of Trem2 caused increased macrophage susceptibility to cholesterol-mediated toxicity and cell death and significantly attenuated atherosclerotic plaque progression in mice.

## Main

Despite recent improvements in cardiovascular disease (CVD) outcomes, CVD remains a leading cause of death^1^. Atherosclerotic plaque formation, a primary cause of CVD, is a disease of the artery wall driven by hyperlipidemia and vascular inflammation^2^. Atherosclerosis is mediated by the deposition of low-density lipoprotein (LDL) cholesterol particles into the arterial intima that accumulate within macrophages, termed foamy cells^3^. These cells constitute a major portion of the total cellularity in early atherosclerotic plaque. Importantly, accumulation of foamy macrophages is associated with increased necrotic core formation and risk of plaque rupture^4^. In early lesions, foamy macrophages derive from a resident pool of aorta intima resident macrophages (Mac^AIR^) but are replaced by recruited monocytes as plaque progresses^5^. In larger lesions, the contribution of monocytes gives way to local macrophage proliferation as a primary mechanism for foamy macrophage maintenance^6^. However, mechanisms regulating foamy macrophage persistence in atherosclerotic lesions are not fully understood^7,8^.

Single-cell RNA sequencing (scRNA-seq) analysis has identified previously unrecognized heterogeneity for macrophage populations within the atherosclerotic aorta^9^. Notably, foamy macrophages have a unique gene signature relative to non-foamy macrophages, including triggering receptor expressed on myeloid cells 2 (Trem2) (refs. ^10–12^). Trem2 is a cell surface lipid sensor that plays a regulatory role in microglia function^13,14^, and polymorphisms are causative for early-onset Alzheimer’s-like dementia^15^. Trem2 signals through the adaptor molecules Dap10/Dap12 to activate Syk, PI3K, AKT and mTOR pathways, activating pro-survival and anti-inflammatory responses^16–19^. Consequently, Trem2 broadly regulates phagocytosis, autophagy, cytoskeletal remodeling and metabolic programming^20,21^. Trem2 promotes homeostatic functions of adipose macrophages, and Trem2 deficiency resulted in enhanced inflammation and adipose hypertrophy^22^. Importantly, Trem2^−/−^ animals have lipid dysregulation and elevated stress hormones, making this mouse difficult to interpret for atherosclerosis^22,23^. Overall, these studies support a role for Trem2 as a lipid sensor and a candidate regulator of foamy macrophage function.

In the present study, we performed trajectory analysis on integrated scRNA-seq data derived from atherosclerotic samples and a genome-wide CRISPR screen to identify Trem2 as a regulator of foamy macrophage differentiation. Macrophage-specific deletion of Trem2 led to reduced macrophage proliferation, enhanced foamy macrophage death and reduced atherosclerotic plaque size. Trem2-deficient foamy macrophages showed an inability to downregulate cholesterol biosynthesis pathways after lipid loading and reduced cell proliferation pathways. This was associated with an upregulated endoplasmic reticulum (ER) stress response, impaired cholesterol efflux and enhanced macrophage cytotoxicity after cholesterol loading. Overall, this study reveals a regulatory module in foamy macrophages reliant on Trem2 for regulating cholesterol accumulation and cell survival and identifies Trem2 as a therapeutic candidate for atherosclerosis.

## Results

### Trem2 is associated with foamy macrophage differentiation

**Fig. 1:**
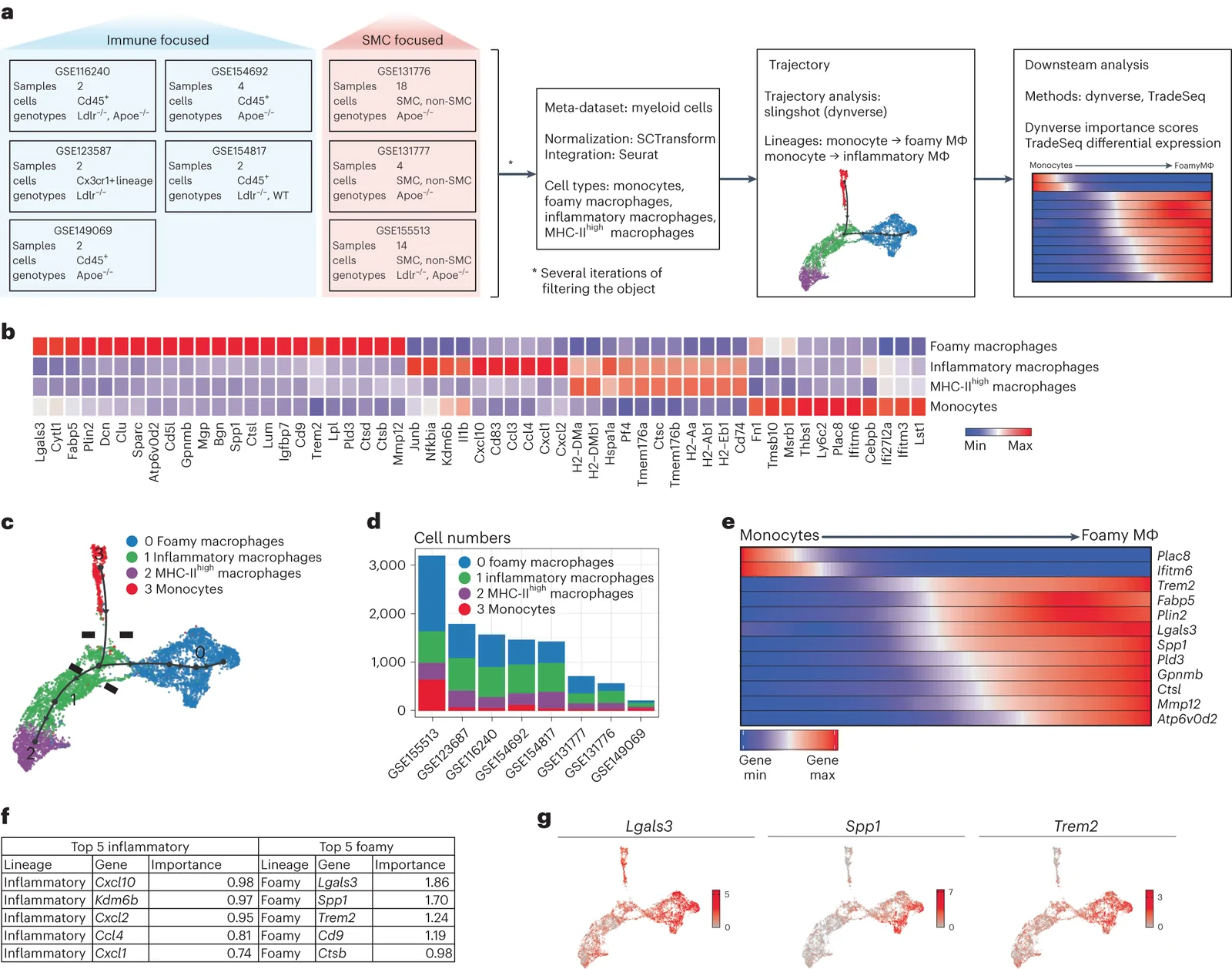
Meta-scRNA-seq trajectory analysis identifies genes associated with foamy macrophage differentiation in atherosclerotic plaques. **a**, scRNA-seq datasets from atherosclerosis studies were integrated into a single meta-dataset. Based on cluster gene enrichment signatures, cells were filtered to isolate intima-associated monocyte and macrophage clusters. Cells were examined for trajectory analysis and differential gene expression. **b**, Four main clusters of intima-associated monocytes/macrophages were identified and annotated based on enriched gene signatures. Top differentially expressed genes are displayed in association with the different clusters. **c**, Trajectory analysis was performed to determine the potential differentiation pathways used by foamy or inflammatory clusters. Data emphasize a monocyte origin and bifurcation toward terminal macrophage differentiation endpoints, with intermediate transition state marked with hash marks. **d**, Monocyte and macrophage cluster representation from original studies is displayed, emphasizing the presence of all clusters from each independent study. **e**, Pseudotime trajectory was plotted between monocyte (cluster 3, origin) and foamy macrophages (cluster 0, endpoint). Genes associated with monocyte lineage, including *Plac8* and *Ifitm6*, were rapidly lost, and genes associated with foamy macrophage specification were enriched across pseudotime. **f**, Trade-seq analysis algorithm predicted genes most likely associated with lineage commitment, called importance index. Top predicted genes for inflammatory and foamy differentiation are outlined in the table. **g**, The top three genes associated with foamy cell ‘importance index’ were plotted on a tSNE project map for gene expression profile. SMC, smooth muscle cell.

**Extended Data Fig. 1:**
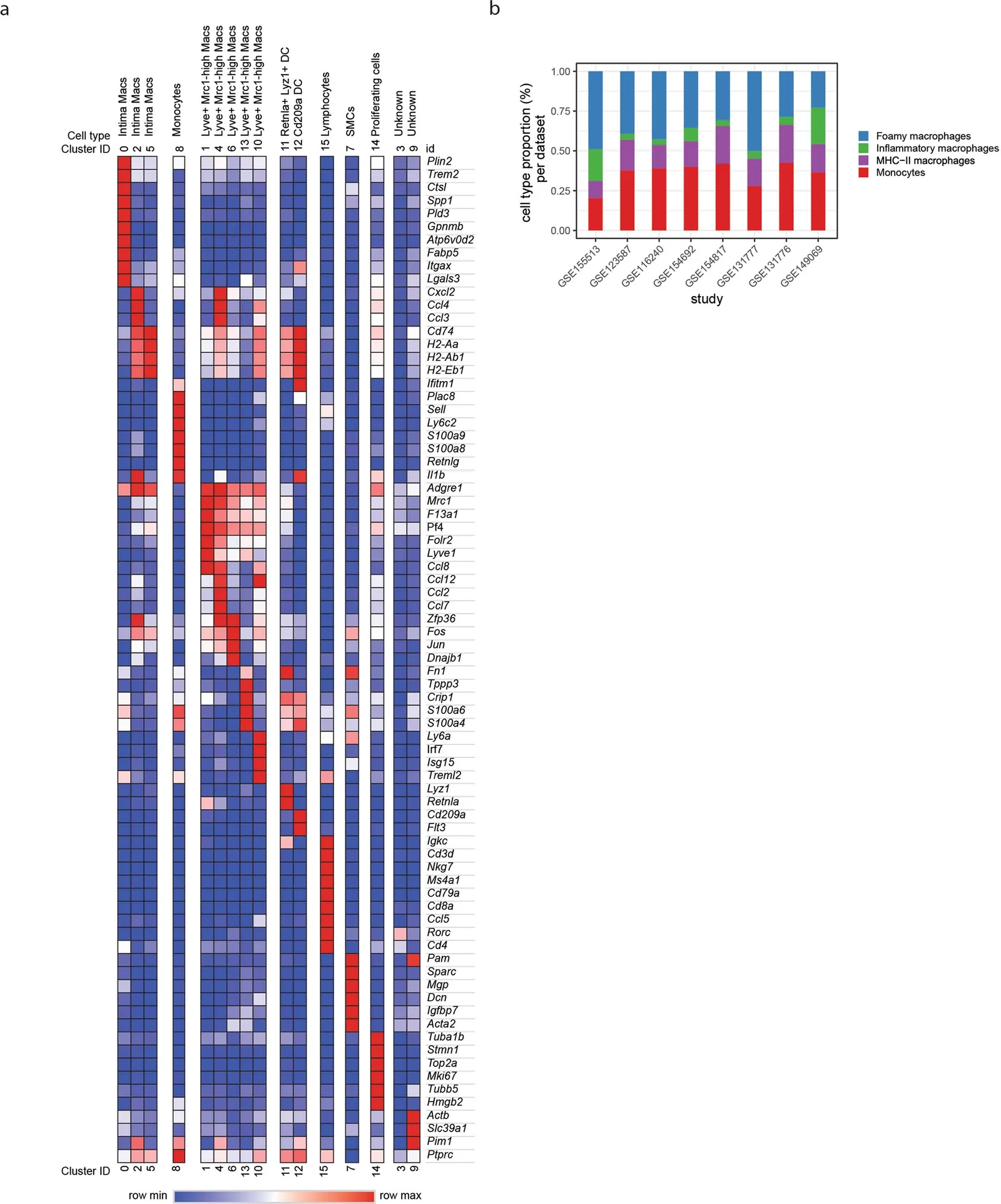
Integrated scRNA-seq differential gene expression. **a**) Heat map of cell clusters identified in META-scRNA-seq dataset, and top enriched genes for each cluster. **b**) Cell type proportion per each dataset from META-scRNA-seq dataset.

Recent efforts in scRNA-seq have generated high-dimensional analysis of immune cell profiles of atherosclerotic plaques, defining previously unknown cell subset heterogeneity^12,24^. However, regulators of fate specification between key subsets remain unclear. We created a meta-dataset of immune cells associated with mouse atherosclerosis using eight publicly available scRNA-seq libraries (Fig. 1a)^5,10,25–29^. Integrated data revealed 16 total clusters, and differential gene expression analysis supported prior meta-analysis studies (Extended Data Fig. 1a)^9^. This included the identification of three intima-associated macrophage populations, a monocyte population and five adventitia macrophage populations. In addition, we identified clusters of dendritic cells (DCs), lymphocytes, proliferating cells as well as smooth muscle cells and two small undefined populations. To remove non-macrophage clusters and clusters not present within atherosclerotic lesions, we used prior sequencing profiles to enrich for monocytes and intima macrophage clusters^10^. The resulting dataset contained four main clusters (Fig. 1b,c), defined by canonical gene markers: monocytes (*Hp*^*+*^*Treml4*^+^*Ly6c2*^+^), foamy macrophages (*Fabp5*^*+*^*Mmp12*^*+*^*Gpnmb*^*+*^*Itgax*^+^*Cd9*^+^), inflammatory macrophages (*Tnf*^*+*^*Nlrp3*^*+*^*Mgl2*^+^*Il1b*^+^) and MHC-II^high^ macrophages (*MHC-II*^+^*Cd74*^+^*H2-DMa*^+^). Inflammatory macrophages shared expression of MHC-II genes with MHC-II^high^ macrophages; however, MHC-II^high^ macrophages lacked an inflammatory cytokine signature. Notably, all intima-associated clusters were represented in each input scRNA-seq dataset, suggesting high reproducibility for cluster identities across studies (Fig. 1d and Extended Data Fig. 1b).

**Extended Data Fig. 2:**
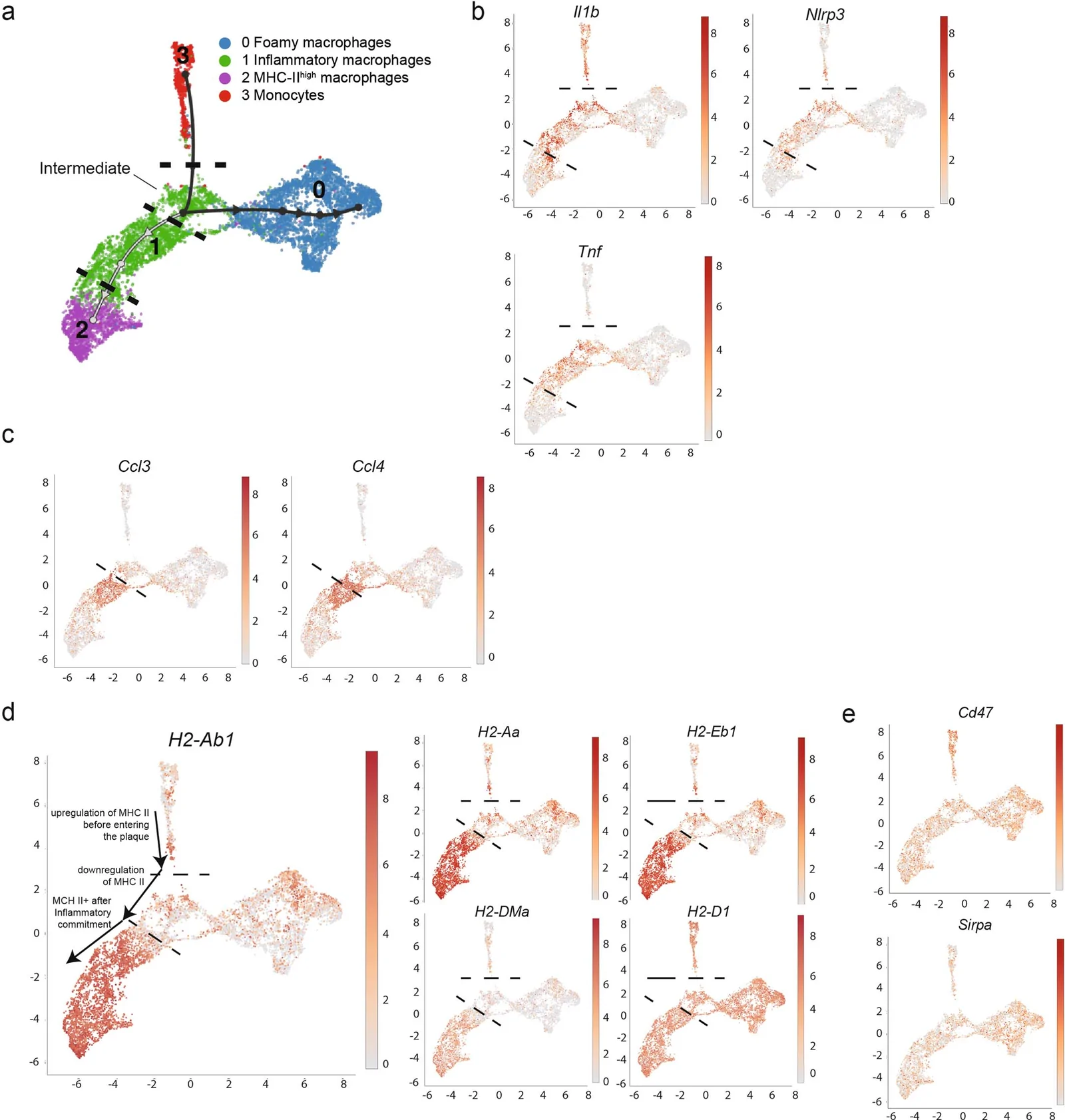
Trajectory analysis of META-scRNA-seq dataset. **a**) Trajectory analysis revealed stages of differentiation following monocyte entry associated with inflammatory macrophage clusters. **b**) Upregulation of inflammation genes associated with entry into intima and commitment toward inflammatory macrophage differentiation. **c**) Ccl3 and Ccl4 were uniquely expressed by committed inflammatory cells, but not by intermediate inflammatory cells. **d**) MHC-II expression is gradually elevated along commitment toward inflammatory differentiation arm of the trajectory map. **e**) Cd47 and Sirpa expression on trajectory map.

To predict differentiation trajectories between intima myeloid cell subsets, we applied the slingshot algorithm from dynverse^30,31^ to our integrated dataset. Using monocytes as the origin, we reveal two trajectories that suggest that binary fate determinates toward either foamy or inflammatory macrophage lineages (Fig. 1c). Surprisingly, trajectory analysis suggests an intermediate inflammatory population shared between differentiation programs, which upregulate modest levels of MHC-II and inflammatory genes before lineage commitment (Fig. 1c and Extended Data Fig. 2a). From this intermediate transition stage, monocytes may differentiate toward foamy lineage, where inflammatory genes are downregulated, or inflammatory lineage, where inflammatory genes are further upregulated (Extended Data Fig. 2b). Although it is difficult to meaningfully split the inflammatory macrophage cluster into several clusters due to the shared continuous expression of canonical inflammatory genes (*Il1b*, *Tnf* and *Nlrp3*), there were genes reserved for further commitment to inflammatory fate, including MHC-II genes, *Ccl3* and *Ccl4* (Extended Data Fig. 2c,d). Interestingly, expression levels of *Sirpa* and *Cd47*, genes associated with efferocytosis, were not differentially expressed between macrophage clusters (Extended Data Fig. 2e).

Next, we sought to visualize the kinetics of the monocyte-to-foamy macrophage differentiation program on pseudotime ordering, showing gene expression changes over pseudotime for selected foamy macrophage genes (Fig. 1e). Although the patterns of activation of foamy macrophage-associated genes *Lgals3*, *Spp1* and *Trem2* are similar, the heat map suggests earlier activation of *Trem2* transcript during lineage commitment. We used dynverse to also obtain gene importance scores associated with each differentiation outcome^30^ (Fig. 1f). Lastly, gene expression plots for top candidate genes, *Lgals3*, *Spp1* and *Trem2*, confirm specificity for the foamy macrophage cell cluster (Fig. 1g). These data identify candidate genes that may be key regulators for differentiation into terminal states. Furthermore, this analysis suggests that monocyte commitment toward fully differentiated plaque macrophages may occur as a binary fate decision from a common inflammatory intermediate population.

**Extended Data Fig. 3:**
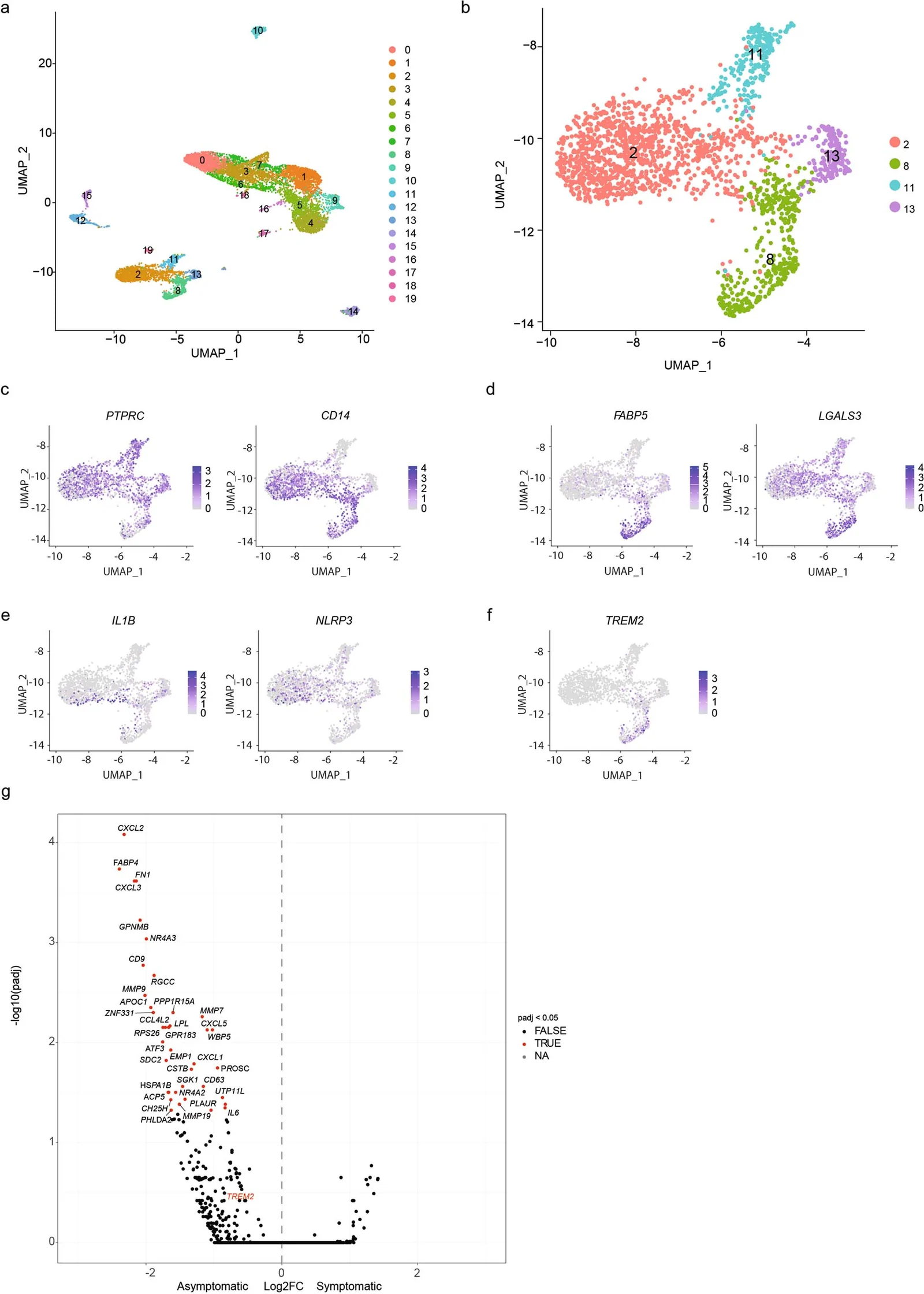
Human atherosclerotic endarterectomy scRNA-seq. **a**) Clustering of all cell subsets from human atherosclerotic endarterectomy samples (Fernandez et. al., Nat Med 2019). **b**) Clustering of monocyte/macrophage populations from human atherosclerotic endarterectomy samples (Fernandez et. al., Nat Med 2019). **c**) PTPRC and CD14 expression of clustered monocyte/macrophage populations from 3b. **d**) Foamy macrophage gene (FABP5, LGALS3) expression of clustered monocyte/macrophage populations from 3b. **e**) Inflammatory macrophage gene (IL1B, NLRP3) expression of clustered monocyte/macrophage populations from 3b. **f**) TREM2 expression of clustered monocyte/macrophage populations from 3b. **g**) Volcano plot of enrichment of genes from samples from either asymptomatic or symptomatic patients. TREM2 in red. DEGs were determined by Wald test with DESeq2.

To compare these findings to human atherosclerosis, we performed characterization of myeloid cells from publicly available symptomatic and asymptomatic human atherosclerotic carotid endarterectomy samples^32^. After data integration, 19 distinct cell populations were identified (Extended Data Fig. 3a). Clustering of monocytes and macrophages generated four unique clusters (Extended Data Fig. 3b) that expressed *PTPRC* and *CD14*, confirming myeloid origins (Extended Data Fig. 3c). Similar to the mouse data, foamy and inflammatory macrophages were distinct populations, with cluster 8 expressing high levels of lipid processing genes (*FABP5* and *LGALS3*), whereas cluster 2 expressed inflammatory genes, including *IL1B* and *NLRP3* (Extended Data Fig. 3d,e). Furthermore, we found that *TREM2* expression was limited to foamy macrophages in human plaques (Extended Data Fig. 3f). Stratification of differentially expressed myeloid genes between asymptomatic and symptomatic plaques found that foamy genes *FABP4*, *CD9* and *LPL* were enriched in asymptomatic plaques (Extended Data Fig. 3g), suggesting that foamy macrophages may promote plaque stability^10^. *TREM2* was also enriched in plaques from asymptomatic patients, but this was not statistically significant (Extended Data Fig. 3g).

### Genome-wide CRISPR knockout screen of oxidized low-density lipoprotein uptake

**Fig. 2:**
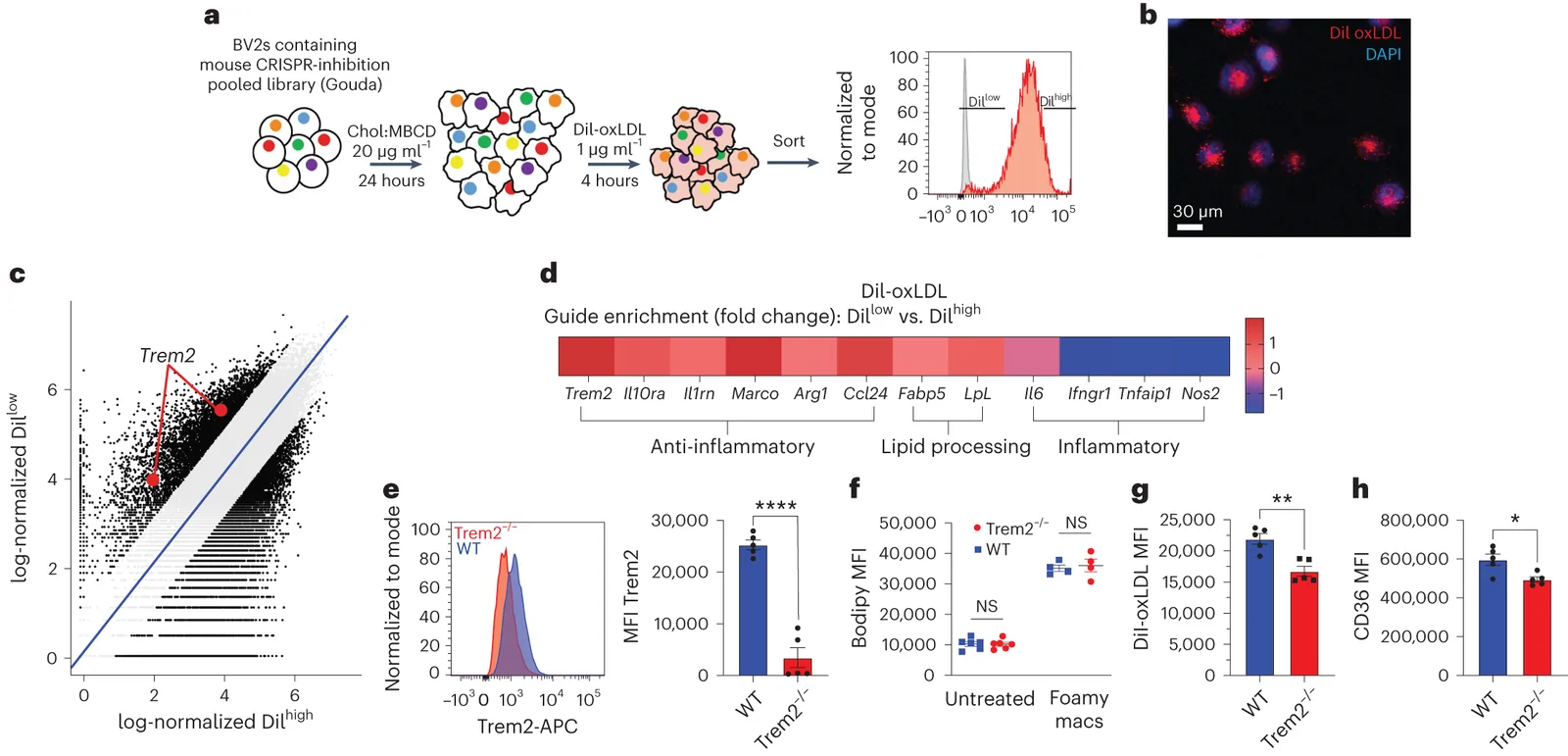
Genome-wide CRISPR screen identifies Trem2 as a candidate regulator for foamy macrophage formation. **a**, Schematic for CRISPR knockout screening approach for oxLDL uptake. BV2 macrophages were loaded with CRISPR pooled guide library (Gouda). Cells were made foamy by overnight treatment with soluble cholesterol and then challenged for 4 h with DiI-oxLDL and sorted for DiI^high^ and DiI^low^ cells. Guides were sequenced from sorted populations. **b**, Confocal micrograph showing BV2 DiI uptake after 4-h incubation with DiI-oxLDL. Representative of two independent experiments. **c**, CRISPR guide enrichment comparing log-normalized enrichment in DiI^high^ (*x* axis) versus DiI^low^ (*y* axis). Gray error bands delineate guides with log fold change < 1. **d**, Selected gene enrichments comparing DiI^low^ versus DiI^high^. **e**, Peritoneal macrophages were isolated from WT or Trem2^−/−^ mice and treated with soluble cholesterol to induce foamy cell formation. After overnight culture, cells were analyzed for Trem2 expression by flow cytometry (*n* = 5 biologically independent replicates per group). Data are mean ± s.e.m. Student’s *t*-test, ****P < 0.0001. **f**, Bodipy staining for total neutral lipid accumulation was performed by flow cytometry on peritoneal macrophages from WT or Trem2^−/−^ mice, cultured overnight in media alone or in media with soluble cholesterol (*n* = 6 biologically independent replicates for untreated and *n* = 4 biologically independent replicates for foamy). Data are mean ± s.e.m. Student’s *t*-test. **g**, Peritoneal macrophages were isolated from WT or Trem2^−/−^ mice and treated with soluble cholesterol to induce foamy cell formation. After overnight culture, cells were treated with DiI-oxLDL for 4 h and assessed for uptake by flow cytometry (*n* = 5 biological replicates per group). Data are mean ± s.e.m. Student’s *t*-test, **P < 0.01. **h**, CD36 expression from peritoneal macrophages isolated from WT or Trem2^−/−^ mice and treated with soluble cholesterol overnight to induce foamy cell formation (*n* = 5 biological replicates per group). Data are mean ± s.e.m. Student’s *t*-test, *P < 0.05. NS, not significant. Source data

**Extended Data Fig. 4:**
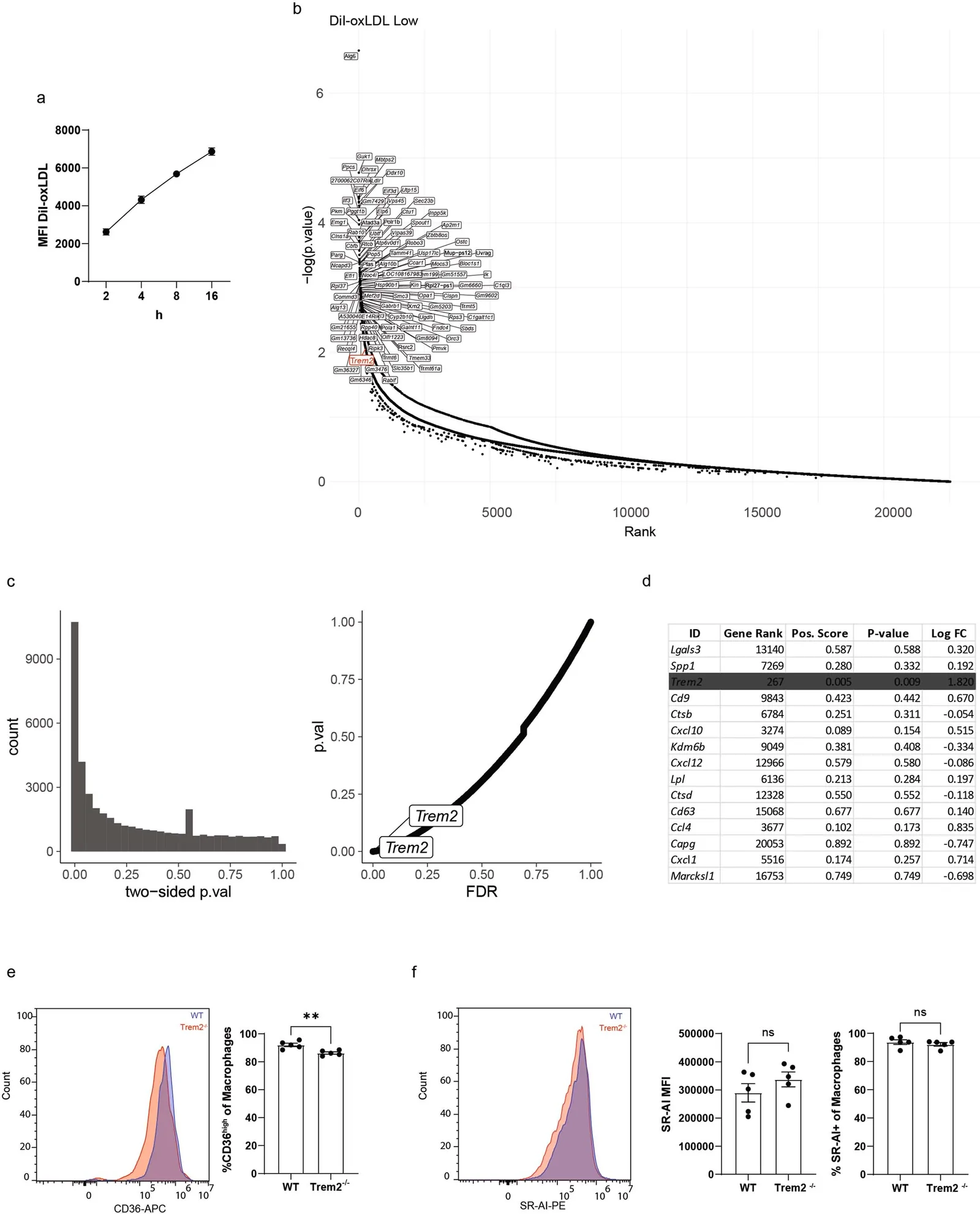
CRISPR screen of foamy macrophage oxLDL uptake and scavenger receptor expression. **a**) Time course analysis of DiI-oxLDL uptake in WT BV2 cells differentiated in media with 20 µg/mL of soluble cholesterol overnight prior to addition of DiI-oxLDL (n = 5 biological replicates/group). Data are mean ± S.E.M. **b**) CRISPR guide enrichment by rank-order was plotted against P-value for DiI-oxLDL-low compared against DiI-oxLDL-high to identify top enriched guides. Trem2 in red. P-values calculated using the negative-binomial model from MAGeCK package and adjusted using Benjamini-Hochberg procedure. **c**) Two sided P-value vs count and p value vs false discovery rate (FDR) for DiI-oxLDL-low compared against DiI-oxLDL-high to identify top enriched guides. p-values and FDR calculated using the negative-binomial model from MAGeCK package and adjusted using Benjamini-Hochberg procedure. **d**) Top 15 ‘importance index’ genes associated with foamy cell commitment by Trade-seq analysis (Fig. 1), were compared for gene rank and enrichment in CRISPR screen. Trem2 highlighted in gray. P-values calculated using the negative-binomial model from MAGeCK package and adjusted using Benjamini-Hochberg procedure. e) CD36 expression (left) and percent CD36 high (right) of foamy peritoneal macrophages cultured with soluble cholesterol overnight from WT (blue) and Trem2-/- mice (red). Gated on F4/80+ CD11b+ live cells (n = 5 biological replicates/group). Data are mean ± S.E.M. Student’s t-test, P = ** < 0.01. **f**) SR-AI expression (left), MFI (middle) and percent SR-AI positive (right) of foamy peritoneal macrophages cultured with soluble cholesterol overnight from WT (blue) and Trem2-/- mice (red). Gated on F4/80+ CD11b+ live cells (n = 5 biological replicates/group). Data are mean ± S.E.M. Student’s t-test Source data

Single-cell trajectory and differential gene expression analysis provided a detailed map of transcriptional changes that occur during foamy macrophage differentiation. However, it is unable to define which genes regulate foamy macrophage differentiation. To determine whether genes expressed during foamy macrophage commitment could also influence the ability of macrophages to accumulate oxidized low-density lipoprotein (oxLDL), we designed an in vitro CRISPR screening approach. For this screen, we elected to differentiate cells into foamy macrophages to mimic macrophages in atherosclerotic plaque. We inserted Cas9 and the ‘Gouda’ knockout pooled CRISPR guide library into the BV2 myeloid cell line^33^. This cell line was selected based on infection efficiency of the library (>90%)^34,35^. To screen for genes associated with oxLDL uptake, cells were loaded with soluble cholesterol and then pulsed with fluorescently labeled DiI-conjugated oxLDL particles (Fig. 2a,b). After 4 h, cells were collected and separated into DiI^low^ or DiI^high^ populations by fluorescence-activated cell sorting (FACS). The 4-h timepoint was selected for the screen because it was within the maximal DiI-oxLDL uptake phase (Extended Data Fig. 4a). The top and bottom 9% of cells labeling with DiI were sorted and sequenced for guide enrichment. Differential guide analysis between DiI^low^ versus DiI^high^ cells revealed gene targets associated with enhanced or reduced oxLDL uptake, including *Trem2* (Fig. 2c and Supplementary Table 1). Unbiased analysis of guide enrichment rank ordered against *P* values and false discovery rate (FDR) are shown (Extended Data Fig. 4b,c). Selected genes associated with lipid processing, classical activation or alternative activation pathways are also shown (Fig. 2d). As expected, loss of lipid processing genes, such as *Lpl* or *Fabp5*, led to reduced ability to take up DiI-oxLDL. Furthermore, anti-inflammatory genes also were enriched in DiI^low^ cells, suggesting that these genes were associated with foamy macrophage maintenance. Interestingly, of the top 15 ‘importance index’ genes identified in foamy cell trajectory analysis in Fig. 1, *Trem2* was the top enriched gene associated with the regulation of oxLDL uptake (Extended Data Fig. 4d).

**Extended Data Fig. 5:**
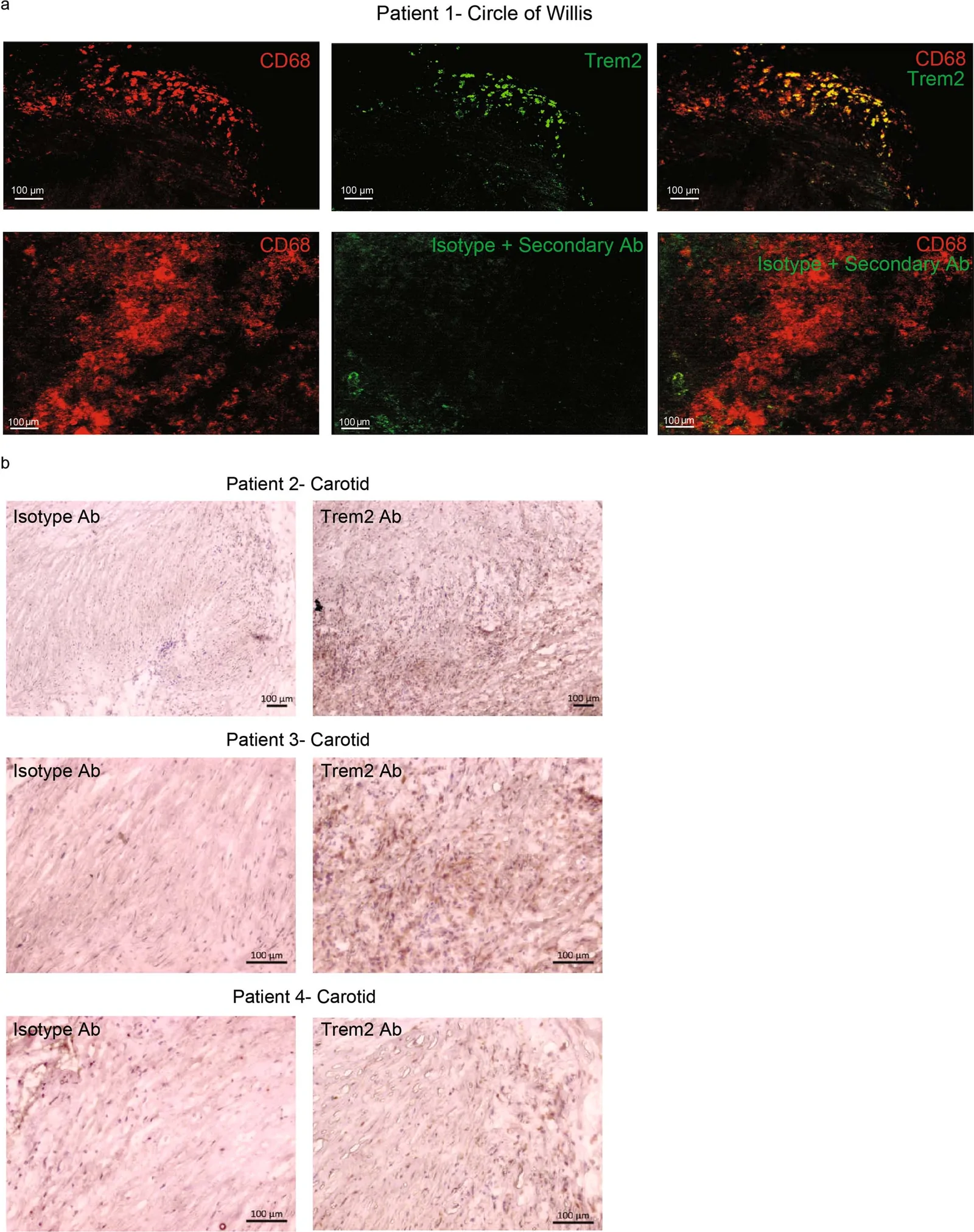
Trem2 staining of human atherosclerotic plaques. **a**) Cranial artery plaques were stained either for CD68 and Trem2 to identify co−expressing foamy macrophages within human plaques (top) or with CD68 and isotype control with secondary antibody (bottom). Representative image from 3 independent samples. **b**) Carotid artery endarterectomy samples from three patients stained for isotype control or Trem2 using DAB (3,3′-Diaminobenzidine) immunohistochemical staining. Representative images from 6 independent samples.

Next, we sought to validate the CRISPR screen result using ex vivo cultured primary macrophages. Peritoneal macrophages were isolated from C57Bl/6 (wild-type (WT)) or Trem2^−/−^ mice and treated with soluble cholesterol overnight. Peritoneal macrophages induced Trem2 expression on WT peritoneal macrophages after cholesterol loading (Fig. 2e), and Bodipy staining for total neutral lipids confirmed similar lipid accumulation in WT and Trem2^−/−^ macrophages (Fig. 2f). However, DiI-oxLDL treatment resulted in reduced fluorescence in Trem2^−/−^ foamy macrophages compared to WT controls (Fig. 2g), confirming that foamy macrophages depend on Trem2 for efficient oxLDL uptake. Furthermore, Trem2^−/−^ cells had decreased expression of CD36 (Fig. 2h and Extended Data Fig. 4e), suggesting that Trem2 signaling may drive oxLDL uptake through regulation of scavenger receptors. We also assessed SR-AI expression, a class A scavenger receptor that mediates LDL uptake, and found no difference in expression (Extended Data Fig. 4f). These data confirm that Trem2 regulates the ability of lipid-loaded macrophages to take up additional oxLDL. Lastly, to confirm the previous findings of *TREM2* expression by human plaque macrophages (Extended Data Fig. 3)^32^, we also confirmed, by protein immunofluorescence, that TREM2 protein was expressed by macrophages present in human carotid plaque (Extended Data Fig. 5). Thus, Trem2 is expressed by plaque-associated macrophages in both mice and humans and is a putative regulator of foamy macrophage formation.

### Trem2 is required for foamy cell formation in vivo

**Fig. 3:**
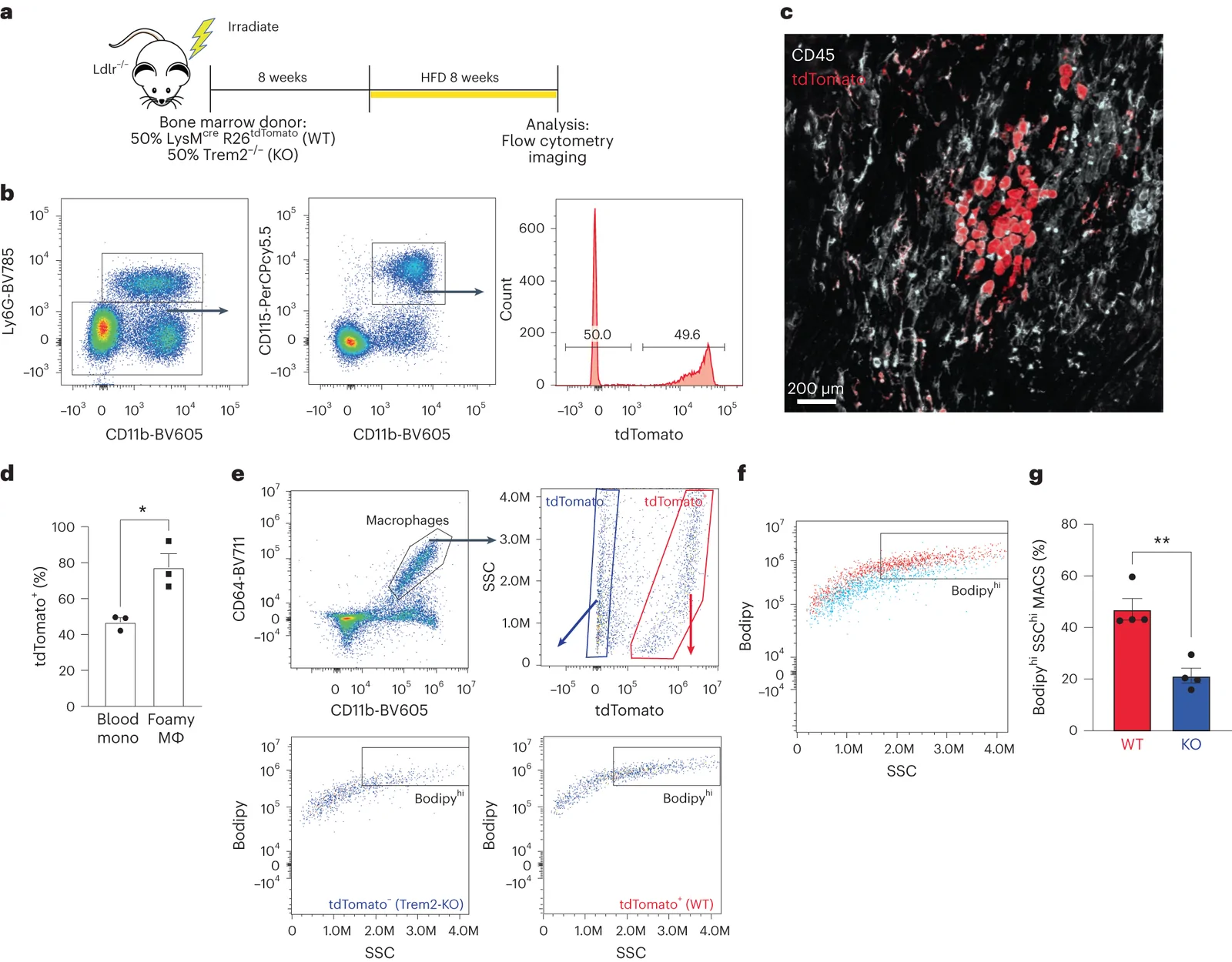
Trem2-deficient macrophages are outcompeted by WT macrophages to form foamy cells in atherosclerotic plaque. **a**, Schematic for mixed bone marrow chimera experiment. Ldlr^−/−^ mice were lethally irradiated and rescued by donor bone marrow from (50%) LysM^cre^ R26^tdTomato^ (WT) and (50%) Trem2^−/−^ mice. Recipient mice were rested for 8 weeks and then fed an HFD for an additional 8 weeks to induce atherosclerosis. **b**, Flow cytometry gating of blood immune cells (CD45^+^) after 8-week HFD feeding, showing ratio of monocytes derived from WT (tdTomato^+^) and Trem2^−/−^ progenitors. **c**, Confocal micrograph of whole-mount aorta showing tdTomato labeling (red) and CD45 (white) staining to define cellular contributions to foamy macrophages. Representative image from two independent experiments. **d**, Quantification of tdTomato^+^ cells in blood compared to foamy macrophages from whole-mount aorta images (*n* = 3 mice per group). Data are mean ± s.e.m. Student’s *t*-test, *P < 0.05. **e**, Foamy FACS was performed on CD64^+^CD11b^+^ macrophages isolated from mixed bone marrow chimera aorta. Macrophages were separated into tdTomato^+^ and tdTomato^−^ populations and then assessed for foamy representation by SSC and Bodipy (neutral lipid) staining. **f**, Flow cytometric overlap between tdTomato^+^ (red) and Trem2^−/−^ (blue) derived macrophages from digested atherosclerotic aorta. **g**, Quantification derived from flow cytometric foamy FACS comparing relative contribution to foamy macrophages (*n* = 4 mice per group). Data are mean ± s.e.m. Student’s *t*-test, **P < 0.01. KO, knockout. MACS, macrophage. Source data

Trem2 regulates macrophage polarization, phagocytosis and survival, but its role in atherosclerosis remains to be examined. Because Trem2^−/−^ mice have elevated cholesterol levels compared to control mice after high-fat diet (HFD) feeding, we elected to use a mixed bone marrow chimera approach to normalize cholesterol levels between strains and allow for examination between WT and Trem2^−/−^ macrophages. Atherosclerosis-susceptible Ldlr^−/−^ mice were lethally irradiated and then transplanted with a 50/50 mix of Trem2^−/−^ bone marrow cells and control bone marrow cells that express LysM^cre^ R26^tdTomato^ reporter allele. Mice were rested for 8 weeks after irradiation, and then chimeric mice were fed an HFD for 8 weeks to induce plaque formation (Fig. 3a). Blood analysis confirmed efficient mixing of Trem2^−/−^ (tdTomato^−^) and control (tdTomato^+^) monocytes in chimeric mice (Fig. 3b). Confocal analysis of the aortic plaques revealed a distinct enrichment of tdTomato^+^ labeling in cells resembling foamy morphology (large and bloated) that co-stained for CD45 (white). Conversely, CD45^+^tdTomato^−^ cells were associated with smaller, non-foamy morphology (Fig. 3c). For quantification, foamy macrophages were blindly identified using morphology and CD68 staining and then separated into tdTomato^+^ (WT) or tdTomato^−^ (Trem2^−/−^) populations. Analysis revealed equal contributions to blood monocytes, but Trem2^−/−^ macrophages failed to compete against WT macrophages to differentiate into foamy macrophages in plaques (Fig. 3d). To confirm this finding, we also employed a ‘Foam FACS’ approach to determine foamy macrophage formation by flow cytometry in mixed chimera mice^10^. After HFD feeding, aortas were isolated and enzymatically digested to liberate macrophages for flow cytometry. Macrophages were identified (CD64^+^CD11b^+^) and then separated into Trem2^−/−^ (tdTomato^−^) or control (tdTomato^+^), and cells were assessed for lipid content (Bodipy) and side scatter (SSC) (Fig. 3e). Control macrophages were more effective at taking up lipid compared to Trem2^−/−^ macrophages (Fig. 3f), and a larger percentage of tdTomato^+^ cells were phenotypically foamy (Fig. 3g). Together, these data support that Trem2 promotes the formation of foamy macrophages in atherosclerosis.

### Loss of Trem2 on macrophages attenuates atherosclerosis progression

**Fig. 4:**
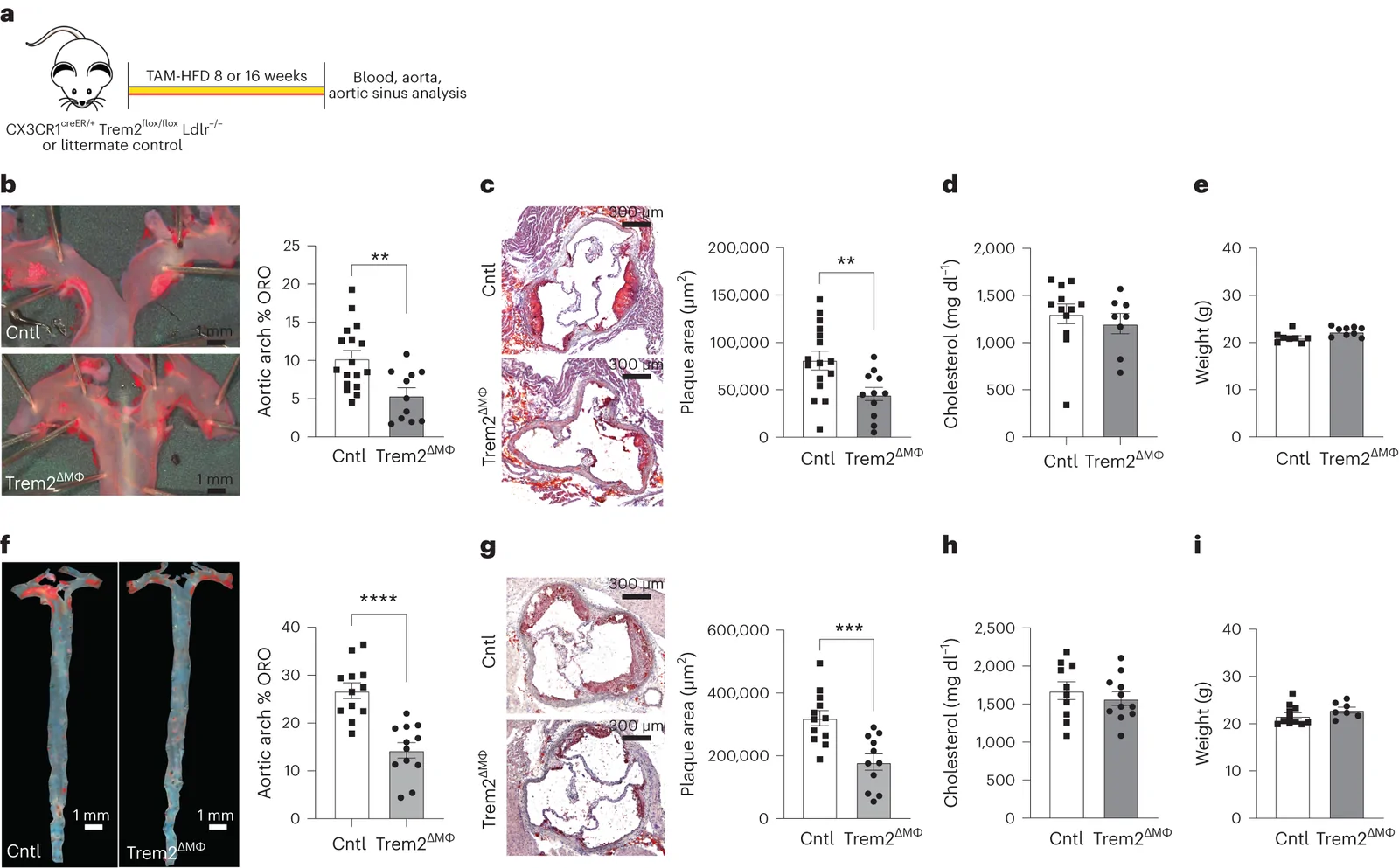
Conditional deletion of Trem2 on macrophages attenuates atherosclerotic plaque progression. **a**, CX3CR1^creER^Trem2^flox/flox^Ldlr^−/−^ (Trem2^ΔMФ^) or littermate control mice (which included Cre^−^ animals CX3CR1^+/+^Trem2^fl/fl^Ldlr^−/−^ and Cre^+^ animals CX3CR1^creER/+^Trem2^fl/+^Ldlr^−/−^) were fed TAM-HFD for 8 weeks (**b**–**e**) or 16 weeks (**f**–**i**). **b**, After 8 weeks of TAM-HFD, aortas were analyzed by en face analysis for percentage Oil Red O (ORO) staining on the arch (*n* = 17 mice per group for Cntl and *n* = 12 for Trem2^ΔMФ^). Data are mean ± s.e.m. Student’s *t*-test, **P < 0.01. **c**, Aortic sinus plaque area measured after ORO staining in 8-week TAM-HFD samples (n = 17 mice per group for Cntl and *n* = 12 for Trem2^ΔMФ^). Data are mean ± s.e.m. Student’s *t*-test, **P < 0.01. **d**, Serum cholesterol levels from 8-week TAM-HFD-fed mice (*n* = 11 mice per group for Cntl and *n* = 8 for Trem2^ΔMФ^). Data are mean ± s.e.m. **e**, Weight data from 8-week TAM-HFD-fed mice (*n* = 9 mice pergroup). Data are mean ± s.e.m. **f**, En face ORO staining of aorta after 16-week TAM-HFD feeding (*n* = 12 mice per group). Data are mean ± s.e.m. Student’s *t*-test, ****P < 0.0001. **g**, Aortic sinus plaque area after 16-week TAM-HFD feeding (*n* = 11 mice per group for Cntl and *n* = 12 for Trem2^ΔMФ^). Data are mean ± s.e.m. Student’s *t*-test, ***P < 0.001. **h**, Serum cholesterol after 16-week TAM-HFD feeding (*n* = 10 mice per group). Data are mean ± s.e.m. **i**, Weight of mice after 16-week TAM-HFD feeding (*n* = 10 mice per group for Cntl and *n* = 7 for Trem2^ΔMФ^). Data are mean ± s.e.m. Source data

**Extended Data Fig. 6:**
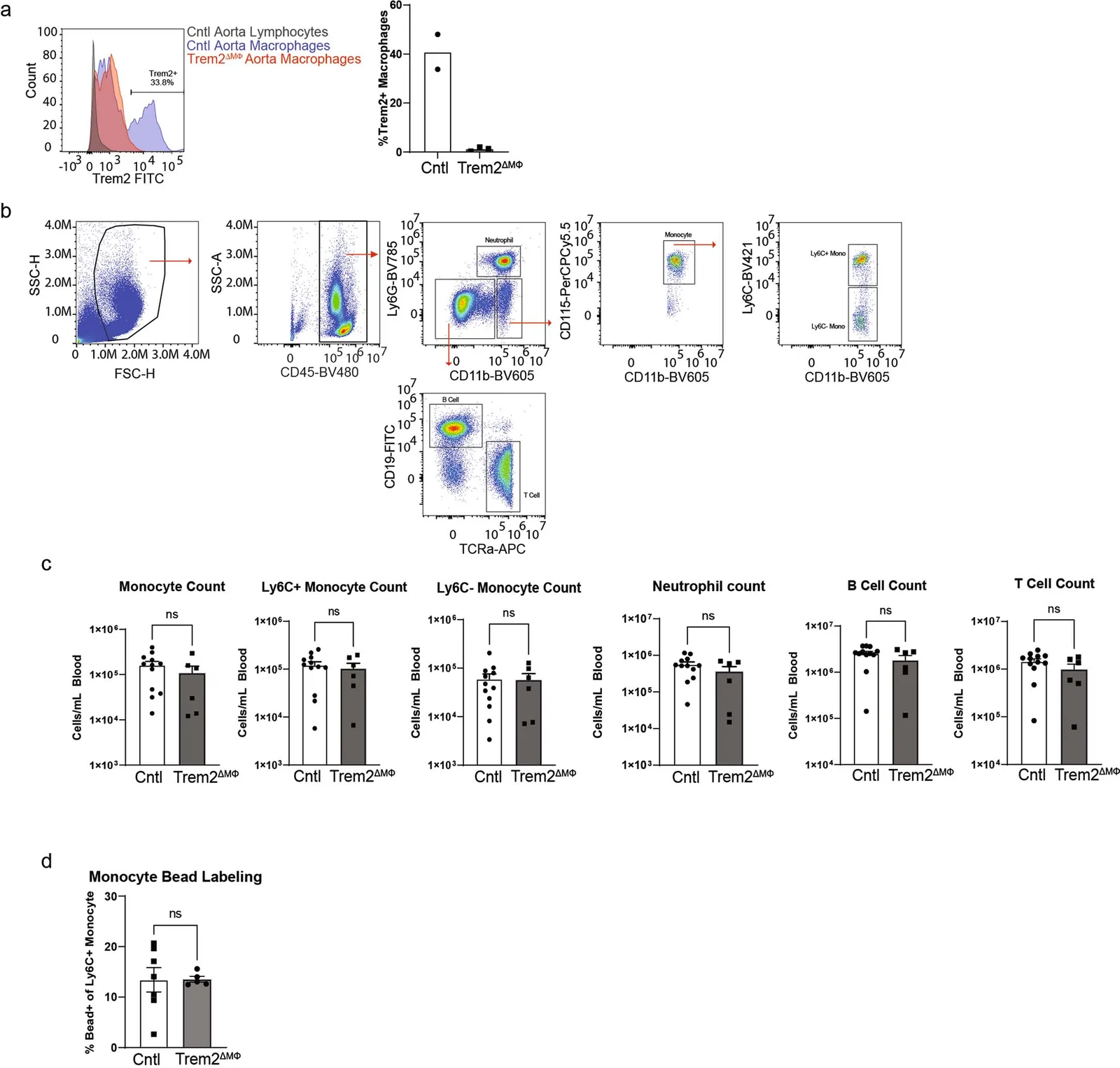
Trem2 deletion and immune profiling in atherosclerotic mice. **a**) Trem2 expression and quantification from atherosclerotic aortae (n = 2 mice/group for Cntl and n = 3 for Trem2ΔMФ). Briefly, Cntl or Trem2ΔMФ mice were fed TAM-HFD for 16 weeks then aorta were harvested, digested and flow cytometry was run. Histogram was gated on live, CD45 + CD11b + CD64+ cells. **b**) Flow cytometric gating strategy for identifying major blood immune cell populations. **c**) Blood immune cell profiling by flow cytometry in indicated mice after 16 weeks TAM-HFD feeding (n = 12 mice/group for Cntl and n = 6 for Trem2ΔMФ). Data are mean ± S.E.M. Student’s t-test. **d**) Classical monocyte bead uptake in the blood was measured by flow cytometry 24 hours after i.v. bead injection in indicated strains after 16 weeks TAM-HFD feeding (n = 7 mice/group for Cntl and n = 5 for Trem2ΔMФ). Data are mean ± S.E.M. Student’s t-test. Source data

Given the systemic defects associated with the Trem2^−/−^ mouse, we crossed Trem2 conditional knockout (Trem2^flox^) mice with CX3CR1^creER-^inducible Cre mice on the Ldlr^−/−^ background (Trem2^ΔMФ^). This approach allows for temporal Trem2 deletion on CX3CR1-expressing cells, which includes all monocytes, plaque-associated macrophages and other CX3CR1-expressing tissue-resident macrophages. Control (Cntl) animals were littermates and included Cre^−^ and Cre^+^ animals; both control strains showed similar results and were combined for reporting. To test the role of Trem2 in plaque formation, Trem2^ΔMФ^ or controls were continuously fed a tamoxifen-enriched HFD (TAM-HFD) for 8 weeks or 16 weeks to induce Trem2 deletion and drive atherosclerosis (Fig. 4a). Deletion of Trem2 in plaque macrophages was confirmed by flow cytometry from aorta after 16-week TAM-HFD feeding (Extended Data Fig. 6a). Strikingly, after 8 weeks of TAM-HFD, atherosclerotic plaques in both the aortic arch and aortic sinus were markedly reduced in the Trem2^ΔMФ^ mice compared to controls (Fig. 4b,c). Notably, this result was independent of changes in serum cholesterol or body weight (Fig. 4d,e). Reduced atherosclerotic plaque formation in the aortic arch and aortic sinus of Trem2^ΔMФ^ mice were replicated after 16 weeks of TAM-HFD feeding (Fig. 4f,g) and was not associated with changes in cholesterol or weight (Fig. 4h,i). Together, these data show that Trem2 is required for atherosclerosis progression and support the hypothesis that Trem2 regulates foamy macrophage formation in atherosclerotic lesions.

### Trem2 regulates foamy macrophage survival in plaque

**Fig. 5:**
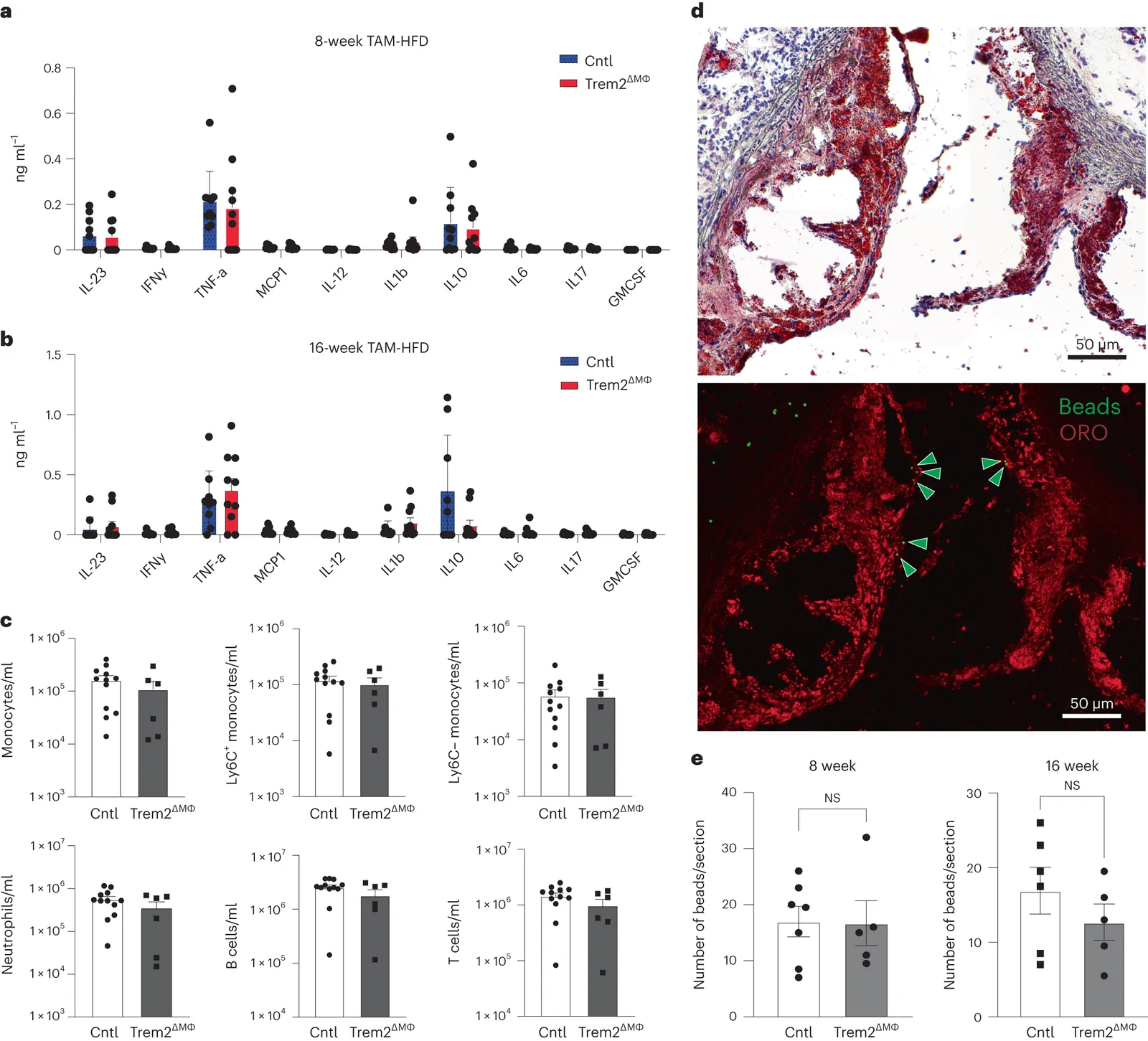
Conditional deletion of Trem2 has no effect on monocyte recruitment or systemic inflammation. **a**, Following the schematic in Fig. 4a, CX3CR1^creER^Trem2^flox/flox^Ldlr^−/−^ (Trem2^ΔMФ^) or littermate control mice were treated continuously with TAM-HFD for the indicated times. **b**, Serum from 8-week TAM-HFD-fed mice were assessed for cytokine levels by multiplex assay (*n* = 10 mice per group). Data are mean ± s.e.m. **c**, Serum from 16-week TAM-HFD-fed mice were assessed for cytokine levels by multiplex assay (*n* = 9 mice per group). Data are mean ± s.e.m. **d**, Blood immune cells were assessed after 16 weeks of TAM-HFD by flow cytometry (*n* = 12 mice per group for Cntl and *n* = 6 for Trem2^ΔMФ^). Data are mean ± s.e.m. **e**, Monocyte recruitment was assessed by bead labeling and recruitment experiment—images from representative histologic and immunofluorescence images with lipid content (red) and beads (green). Representative image from two independent experiments. **f**, Quantification of plaque-associated beads that were counted per section for 8-week or 16-week TAM-HFD experiments from experiments in Fig. 4 (*n* = 7 mice per group for Cntl and *n* = 5 for Trem2^ΔMФ^). Data are mean ± s.e.m. Student’s *t*-test. NS, not significant; ORO, Oil Red O. Source data

To determine mechanisms regulating plaque progression in Trem2^ΔMФ^ mice, we investigated whether there were systemic changes in inflammation. First, by serum cytokine multiplex assay, we observed no significant changes across a panel of 10 cytokines associated with atherosclerosis progression at 8 weeks or 16 weeks of TAM-HFD feeding (Fig. 5a,b). Next, because increased blood monocyte numbers are associated with elevated atherosclerotic plaque formation^36^, we performed flow cytometry to assess peripheral blood immune cell populations (Extended Data Fig. 6b). Data revealed no substantial changes in monocyte or other immune cell numbers in the blood, suggesting that changes in systemic inflammation were not a major driver of the reduced atherosclerosis observed in Trem2^ΔMФ^ mice (Fig. 5c and Extended Data Fig. 6c).

**Extended Data Fig. 7:**
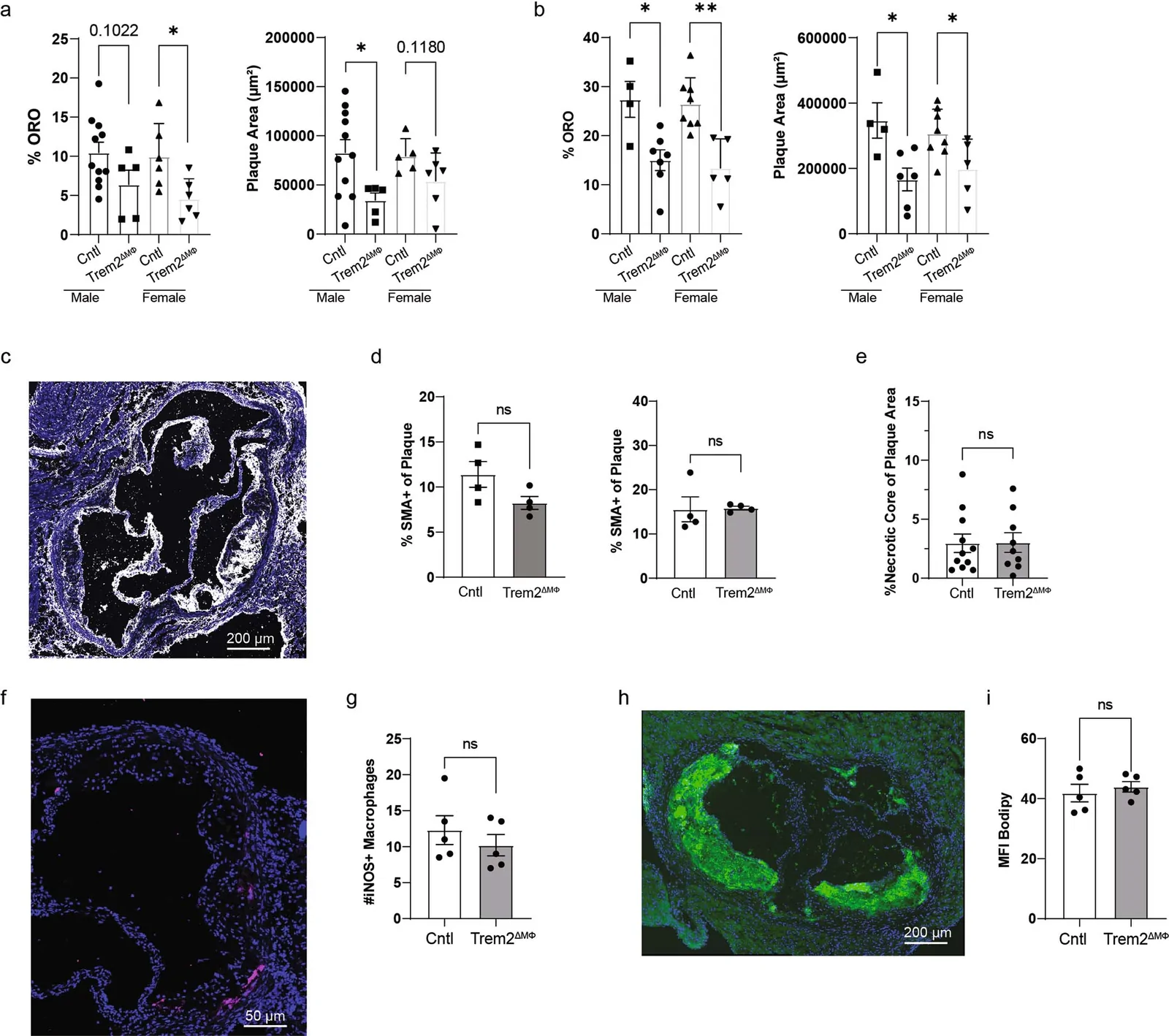
Plaque phenotypes of control or Trem2-deficent mice. **a**) Aortic arch and sinus plaque quantification from 8-week TAM-HFD fed mice split by sex (n = 11 mice/group Cntl male, n = 5 Trem2ΔMФ male, n = 6 Cntl female, n = 6 Trem2ΔMФ female). Data are mean ± S.E.M. Two−tailed ANOVA, P = * < 0.05. **b**) Aortic arch and sinus plaque quantification from 16-week TAM-HFD fed mice split by sex (n = 4 mice/group Cntl male, n = 6 Trem2ΔMФ male, n = 8 Cntl female, n = 5 Trem2ΔMФ female). Data are mean ± S.E.M. Two−tailed ANOVA, P = * < 0.05, **<0.01. **c**) Representative imaging of smooth muscle cells, by SMA staining. **d**) Quantification of SMA staining percentage of plaque at 8 (left) and 16 weeks (right) of TAM-HFD (n = 4 mice/group). Data are mean ± S.E.M. Student’s t-test. **e**) Necrotic core quantification from 16 week TAM-HFD fed Cntl or Trem2ΔMФ (n = 11 mice/group for control and n = 9 for Trem2ΔMФ). Data are mean ± S.E.M. Student’s t-test. **f**) Representative imaging of iNOS+ macrophages (magenta) from Ctrl 16 week TAM-HFD mouse plaque. **g**) Quantification of the number of iNOS+ macrophages (CD68) from Ctrl and Trem2ΔMФ plaques after 16 week TAM-HFD (n = 5 mice/group). Data are mean ± S.E.M. Student’s t-test. **h**) Representative Bodipy staining (green) from Ctrl 16 week TAM-HFD mouse plaque. **i**) Quantification of the mean pixel intensity of Bodipy from Ctrl and Trem2ΔMФ plaques after 16 week TAM-HFD (n = 5 mice/group). Mean pixel intensity was determined via imageJ by outlining the plaque and calculating mean fluorescence intensity. Data are mean ± S.E.M. Student’s t-test. Source data

We next performed a monocyte recruitment assay by labeling monocytes with fluorescent beads to determine whether there were changes in recruitment to lesions after 8 weeks or 16 weeks of TAM-HFD. Following established protocols^37,38^, beads were injected intravenously (i.v.) to label classical monocytes; labeling efficiency was checked after 24 h; and mice were killed to assess monocyte infiltration into lesions 48 h after bead labeling. Figure 5d shows representative plaque area and bead recruitment to lesions. Beads typically infiltrated the surface of lesions, as previously described^38^. Notably, bead uptake by blood monocytes was similar between Cntl and Trem2^ΔMФ^ mice (Extended Data Fig. 6d). Quantification of beads in atherosclerotic lesions revealed similar recruitment rates between experimental groups (Fig. 5e). This was independent of changes in atherosclerotic lesion size. Changes in plaque lesion size were also independent of sex, where male and female mice show similar trends in reduced plaque size when Trem2 was conditionally deleted (Extended Data Fig. 7a,b). Cumulatively, these data suggest that changes in plaque size are likely associated with local changes in foamy macrophage function or persistence in lesions.

**Fig. 6:**
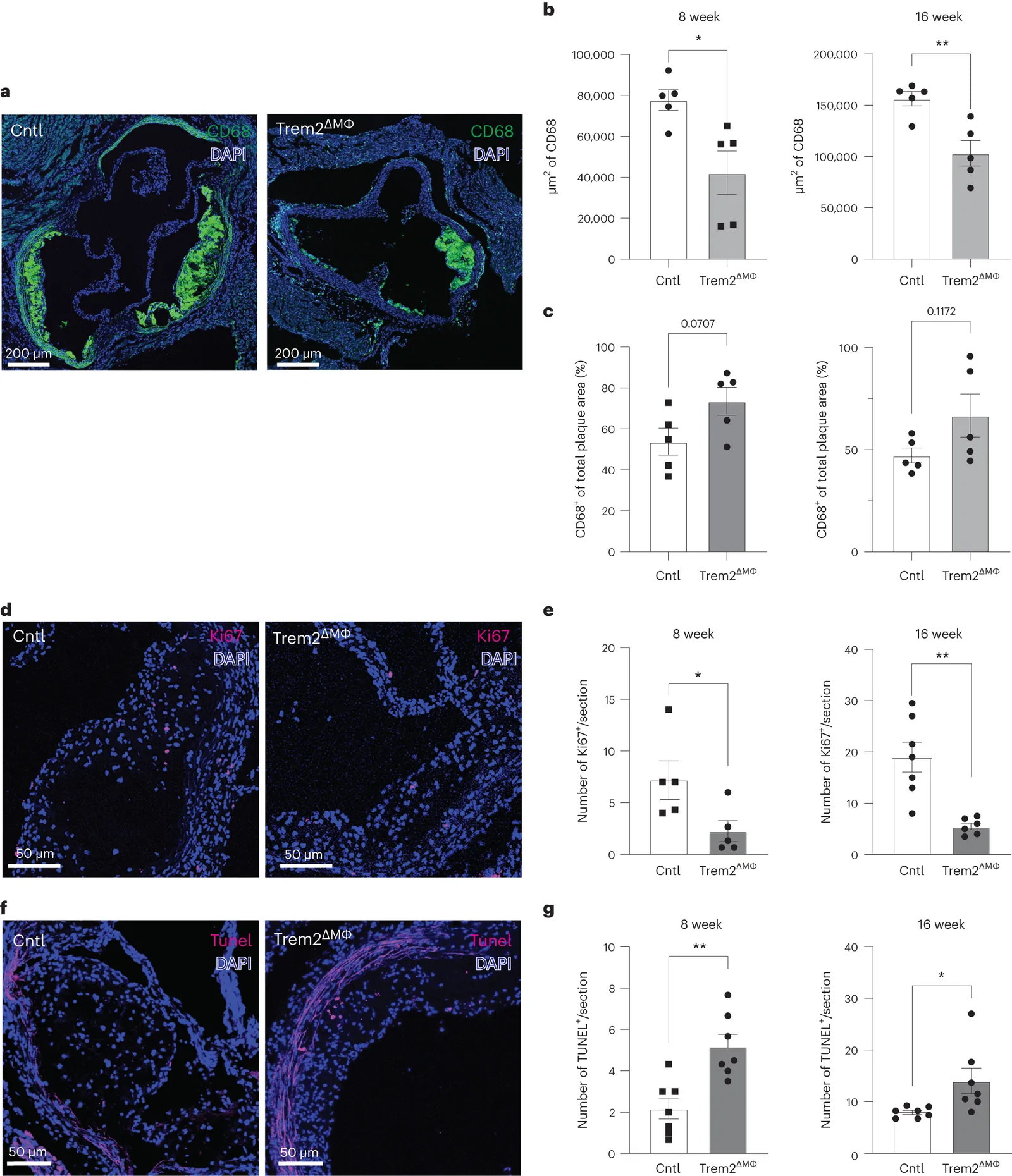
Trem2 regulates foamy macrophage survival and proliferation in atherosclerotic lesions. **a**, Confocal micrograph showing CD68 staining (green) and DAPI (blue) for macrophage area in Cntl or Trem2-deficent mice after 16-week TAM-HFD feeding. Representative image from two independent experiments. **b**, Quantification of CD68^+^ macrophage area per section in 8-week or 16-week TAM-HFD samples (*n* = 5 mice per group). Data are mean ± s.e.m. Student’s *t*-test, *P < 0.05 and **P < 0.01. **c**, Quantification of the percentage of plaque that is macrophages (CD68^+^) in 8-week or 16-week TAM-HFD samples (*n* = 5 mice per group). Data are mean ± s.e.m. Student’s *t*-test. **d**, Confocal micrograph showing Ki67 staining (magenta) and CD68 staining (green) for proliferation in Cntl or Trem2-deficient mice after 16-week TAM-HFD feeding. Representative image from two independent experiments. **e**, Quantification of Ki67^+^ macrophages (CD68^+^) per section in 8-week or 16-week TAM-HFD samples (*n* = 5 mice per group for 8-week TAM-HFD, *n* = 7 mice per group for Cntl 16-week TAM-HFD and *n* = 6 mice per group for 16-week TAM-HFD Trem2^ΔMФ^). Data are mean ± s.e.m. Student’s *t*-test, *P < 0.05 and **P < 0.01. **f**, Confocal micrograph of TUNEL staining (magenta) and CD68 staining (green) for detection of dying cells within atherosclerotic lesions after 16-week TAM-HFD feeding. Representative image from two independent experiments. **g**, Quantification of TUNEL^+^ macrophages (CD68^+^) per section in 8-week or 16-week TAM-HFD samples (*n* = 6 mice per group for Cntl 8-week TAM-HFD, *n* = 7 mice per group for Trem2^ΔMФ^ 8-week TAM-HFD, *n* = 7 mice per group for Cntl 16-week TAM-HFD and *n* = 7 mice per group for Trem2^ΔMФ^ 16-week TAM-HFD). Data are mean ± s.e.m. Student’s *t*-test, *P < 0.05 and **P < 0.01. Source data

To test local plaque changes in TAM-HFD-fed Trem2^ΔMФ^ or Cntl mice, we performed confocal microscopy to assess plaque macrophage and smooth muscle area using immunofluorescence staining of aortic sinus sections. Macrophages were identified using CD68 antibody and smooth muscle cells with alpha actin (SMA) (Fig. 6a and Extended Data Fig. 7c). Quantification confirmed reduced total macrophage area in Trem2^ΔMФ^ mice at 8 weeks and 16 weeks of TAM-HFD (Fig. 6a,b). However, as a percentage of total plaque, macrophage area was trending larger in the Trem2-deficient mice (Fig. 6c), which is consistent with less developed plaques, before the formation of a fibrous cap, smooth muscle-derived foam cells or a necrotic core^39^. SMA-expressing fibrous cap size was further examined, but no difference between groups at either timepoint was observed (Extended Data Fig. 7d). We also assessed necrotic core formation and found no significant differences (Extended Data Fig. 7e).

Because Trem2 is associated with alternative activation responses in macrophages^40^, we next stained sections for iNOS expression to detect whether macrophage activation states were affected in Trem2-deficient plaques. By performing immunofluorescence staining and quantification, we found no differences in the number of iNOS^+^ macrophages after 16 weeks of TAM-HFD feeding (Extended Data Fig. 7f,g), suggesting that Trem2 deletion may not affect pro-inflammatory macrophage skewing within plaques. Moreover, given the reduced number of macrophages within Trem2^ΔMФ^ plaques and that Trem2^−/−^ peritoneal macrophages had decreased ox-LDL uptake, we tested whether there were changes in total lipid content in Trem2-sufficient or Trem2-deficient plaques. We measured total plaque lipid content by staining for neutral lipids (Bodipy) by confocal microscopy and quantified average pixel intensity across lesions (Extended Data Fig. 7h). Data revealed no change in the MFI (mean fluorescence intensity) of Bodipy between Trem2^ΔMФ^ and Ctrl plaques at 16 weeks after TAM-HFD (Extended Data Fig. 7i), suggesting that the reduction in plaque macrophages was not leading to excess accumulation of cholesterol in the lesions of Trem2^ΔMФ^ animals.

Next, to detect potential changes in proliferation, we performed immunostaining for Ki67 (Fig. 6d). Quantification of CD68^+^Ki67^+^ macrophages within lesions showed a marked reduction in local proliferation in Trem2^ΔMФ^ plaques at both 8-week and 16-week TAM-HFD feeding (Fig. 6e). To test whether loss of Trem2 resulted in changes in macrophage susceptibility to death in plaques, we also performed TUNEL staining to identify apoptotic cells (Fig. 6f). TUNEL^+^CD68^+^ macrophages were markedly enriched in Trem2^ΔMФ^ plaques at both timepoints analyzed (Fig. 6g). Together, these data suggest that foamy macrophages rely on Trem2 to persist and proliferate in atherosclerotic lesions.

### Trem2 deletion in established plaque slows atherosclerosis progression

**Fig. 7:**
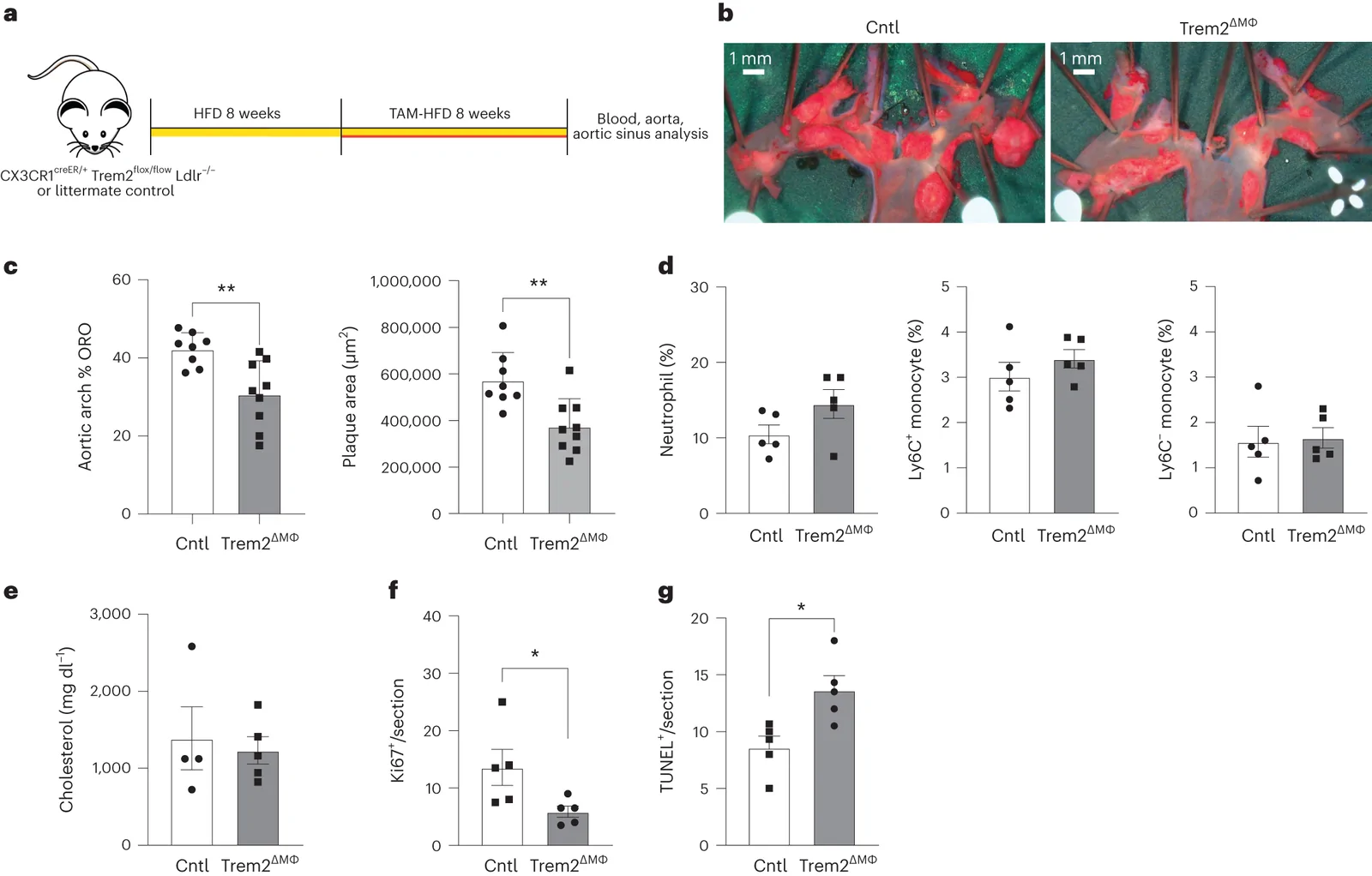
Deletion of Trem2 in established atherosclerotic lesions leads to enhanced foamy macrophage death and reduced atherosclerotic plaque size. **a**, Schematic for intervention study where mice were fed an HFD for 8 weeks and then switched to TAM/HFD for an additional 8 weeks before being killed. **b**, En face aorta analysis of plaque area after 16 weeks of diet-switch intervention study. Representative image from two independent experiments. **c**, Quantification of plaque area in aorta and aortic sinus (*n* = 8 mice per group for Cntl and *n* = 9 for Trem2^ΔMФ^). Data are mean ± s.e.m. Student’s *t*-test, **P < 0.01. **d**, Blood immune population analysis after 16-week diet-switch intervention model (*n* = 5 mice per group). Data are mean ± s.e.m. **e**, Total serum cholesterol levels after 16-week diet-switch model (*n* = 4 mice per group for Cntl and *n* = 5 for Trem2^ΔMФ^). Data are mean ± s.e.m. **f**, Quantification of plaque macrophage proliferation analysis by Ki67^+^ macrophages (CD68^+^) (*n* = 5 mice per group). Data are mean ± s.e.m. Student’s *t*-test, *P < 0.05. **g**, Quantification of TUNEL^+^ macrophages (CD68^+^) in plaques after 16-week diet-switch model (*n* = 5 mice per group). Data are mean ± s.e.m. Student’s *t*-test, *P < 0.05. ORO, Oil Red O. Source data

Because humans are diagnosed with atherosclerosis once plaque has already developed, we wanted to test whether Trem2 can be targeted therapeutically in established atherosclerotic lesions. We designed an in vivo intervention study by feeding Trem2^ΔMФ^ mice or littermate controls a regular HFD for 8 weeks to induce atherosclerotic lesions in all animals and then transitioning the mice to a TAM-HFD to allow for deletion of Trem2 on CX3CR1-expressing cells for an additional 8 weeks. After 16 weeks of total HFD feeding, mice were killed and assessed for atherosclerosis progression (Fig. 7a). Aortic arch and aortic sinus were measured for atherosclerotic plaque area and revealed that myeloid-specific deletion of Trem2 in established lesions attenuated further atherosclerosis progression (Fig. 7b,c). This outcome was independent of blood monocyte numbers or serum cholesterol levels (Fig. 7d,e). Similar to data from the continuous treatment experiments, plaque macrophages from Trem2^ΔMФ^ mice showed reduced Ki67 positivity and elevated TUNEL staining compared to controls (Fig. 7f,g). Overall, these data emphasize the potential for targeting Trem2 to reduce further atherosclerosis progression in patients with established plaques.

### Trem2 regulates foamy macrophage cholesterol sensing and ER stress

**Fig. 8:**
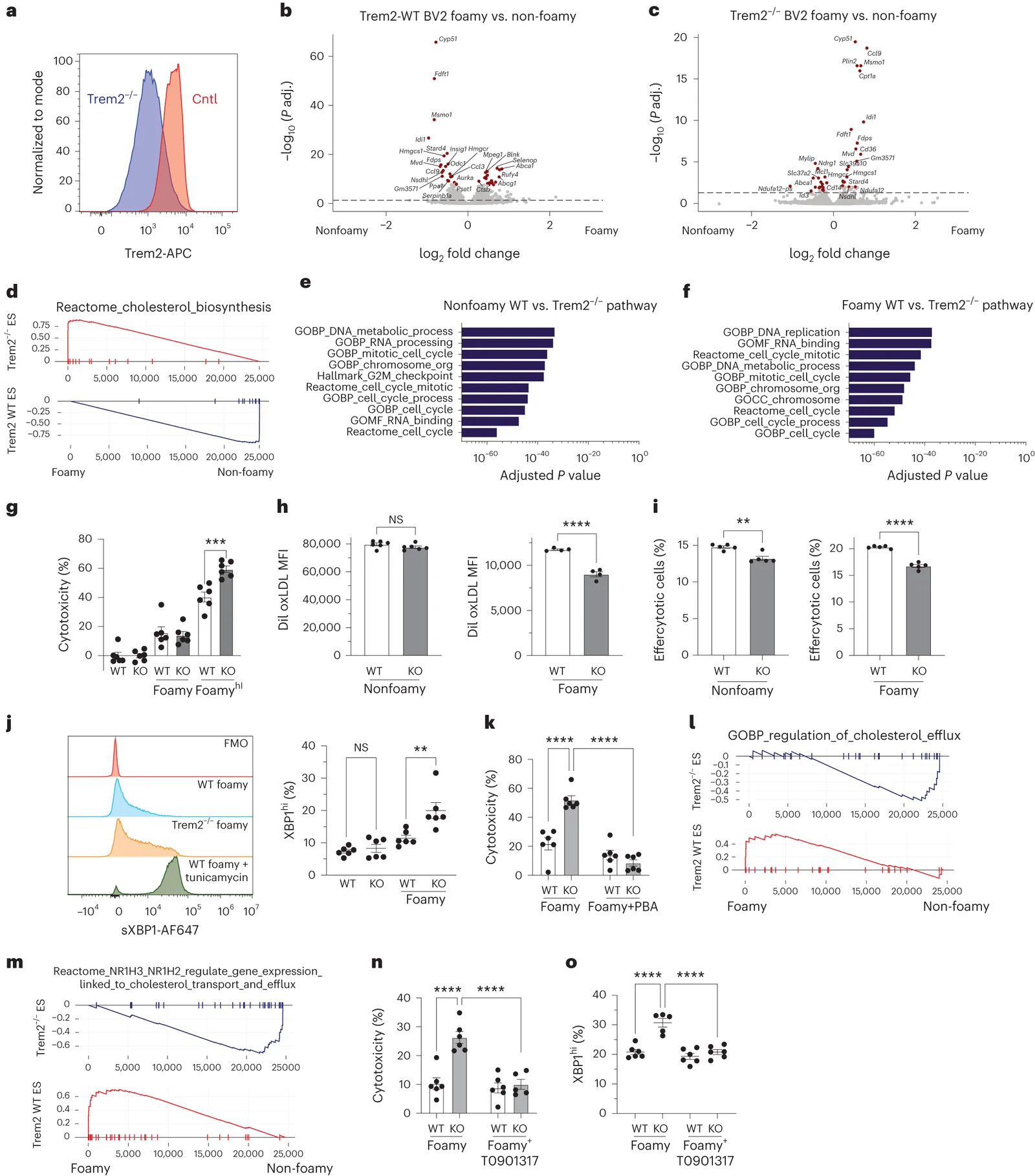
Trem2-deficient foamy macrophages are susceptible to cell death and enhanced ER stress response through dysfunctional LXR signaling. **a**, WT and Trem2^−/−^ BV2s assessed for Trem2 expression by flow cytometry. WT (**b**) or Trem2^−/−^ (**c**) BV2 macrophages DEGs from bulk RNA-seq determined by Wald test with DESeq2. **d**, GSEA plot of cholesterol biosynthesis pathways. ES, enrichment score. **e**, Pathway analysis of RNA-seq data comparing WT and Trem2^−/−^ non-foamy BV2 cells. **f**, Pathway analysis of RNA-seq data comparing WT and Trem2^−/−^ foamy BV2 cells. **e**,**f**, Significant pathways determined using weighted Kolmogorov–Smirnov test. **g**, WT or Trem2^−/−^ cell supernatant assessed for cytotoxicity by LDH assay after 16 h (*n* = 6 biological replicates per group). Foamy: 20 µg ml^−1^ cholesterol; foamy^hi^: 80 µg ml^−1^ cholesterol. Data are mean ± s.e.m. Two-tailed ANOVA, ***P < 0.001. **h**, DiI-oxLDL uptake for WT or Trem2^−/−^ non-foamy and foamy BV2 macrophages (*n* = 6 for non-foamy WT and Trem2^−/−^ and *n* = 4 foamy WT and Trem2^−/−^ biological replicates). Data are mean ± s.e.m. Student’s *t*-test, ***P < 0.001. **i**, WT or Trem2^−/−^ non-foamy and foamy BV2 macrophage efferocytosis. Efferocytotic cells were determined by the percent of BV2s that were positive for CTV-labeled splenocytes (*n* = 5 biological replicates per group). Data are mean ± s.e.m. Student’s *t*-test, **P < 0.01 and ****P < 0.0001. **j**, WT or Trem2^−/−^ non-foamy and foamy BV2 macrophage sXBP1 expression. Tunicamycin was used as a positive control (*n* = 6 biological replicates per group). FMO (fluorescence minus one) shows unstained control. Data are mean ± s.e.m. Two-tailed ANOVA, **P < 0.01. **k**, WT or Trem2^−/−^ foamy BV2 macrophages (80 µg ml^−1^ cholesterol) plus 10 µM PBA. Cell supernatant was assessed for cytotoxicity by LDH assay after 16 h (*n* = 6 biological replicates per group). Data are mean ± s.e.m. Two-tailed ANOVA, ****P < 0.0001. **l**, GSEA plot of cholesterol efflux pathways from RNA-seq. **m**, GSEA plot of NR1H2 and NR1H3 gene target pathways from RNA-seq. **n**, WT or Trem2^−/−^ foamy BV2 macrophages (80 µg ml^−1^ cholesterol) ± T0901317 percent cytotoxicity (*n* = 5 biological replicates per group). Data are mean ± s.e.m. Two-tailed ANOVA, ****P < 0.0001. **o**, WT or Trem2^−/−^ foamy BV2 macrophages (80 µg ml^−1^ cholesterol) ± 10 µM T0901317, assessed for sXBP1 levels by flow cytometry. Tunicamycin was used as a positive control (*n* = 5 biological replicates per group). Data are mean ± s.e.m. Two-tailed ANOVA, ****P < 0.0001. KO, knockout; NS, not significant. Source data

**Extended Data Fig. 8:**
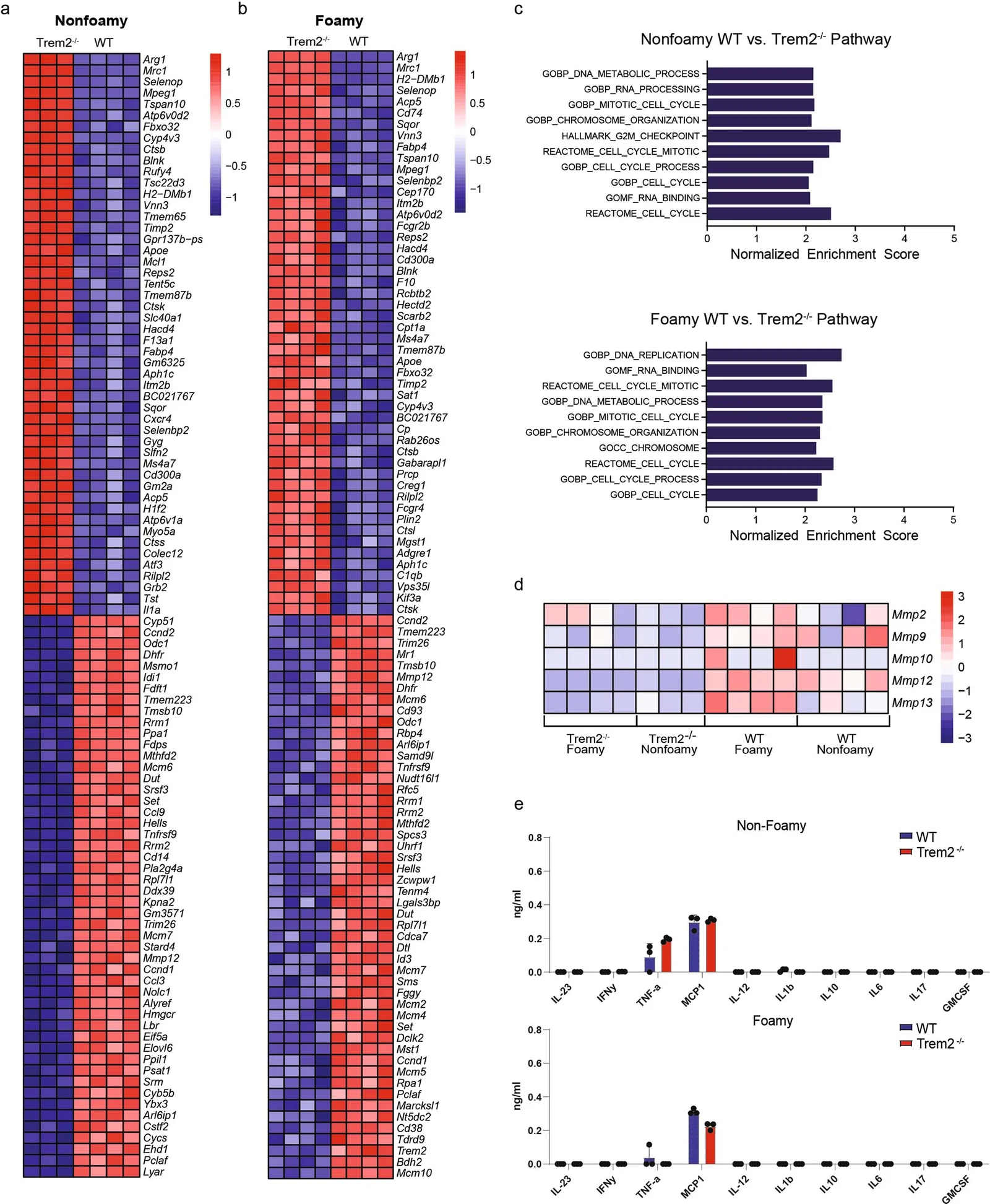
Bulk RNA-seq and cytokine production analysis of WT or Trem2^−/−^ BV2 cells. **a**) Heat map of nonfoamy macrophages comparing top enriched WT and Trem2-/- genes. **b**) Heat map of foamy macrophages comparing top enriched WT and Trem2-/- genes. **c**) Normalized enrichment scores for top pathways associated with WT BV2 compared to Trem2-/- BV2 in media alone (nonfoamy) or following foamy differentiation. Significant pathways were determined using Weighted-Kolmogorov-Smirnov (WKS) test. **d**) Heat map of nonfoamy and foamy macrophages comparing matrix metalloprotease genes from WT and Trem2-/- BV2. **e**) Cytokine supernatant analysis from cultured WT or Trem2-/- cells cultured with either media alone or media with 20 mg/ml soluble cholesterol overnight (n = 3 biological replicates/group). Data are mean ±S.E.M. Source data

To examine the mechanisms that are regulated downstream of Trem2 in foamy macrophages, we generated a Trem2^−/−^ BV2 macrophage cell line to allow for rapid assessment of the role of Trem2 in a homogenous cell line. For in vitro studies, BV2 cells were cultured in media alone, in soluble cholesterol at 20 µg ml^−1^ or at ‘high’ dosing at 80 µg ml^−1^. First, we validated that we had CRISPR–Cas9 knockout of Trem2 protein in BV2 cells after soluble cholesterol treatment (Fig. 8a). We next sought to understand the molecular regulation of Trem2 in non-foamy and foamy macrophages. Thus, we performed bulk RNA-seq analysis of Cntl or Trem2^−/−^ BV2 cells treated overnight in media alone or in 20 µg ml^−1^ soluble cholesterol. We were interested in understanding the response to cholesterol loading, so we compared WT BV2 to WT foamy and Trem2^−/−^ BV2 to Trem2^−/−^ foamy. As expected, WT foamy cells showed a strong upregulation of cholesterol efflux genes (*Abca1* and *Abcg1*) and a downregulation of cholesterol synthesis genes (*Cyp51* and *Hmgcr*) (Fig. 8b). Surprisingly, these features were inversely associated in the Trem2^−/−^ foamy cells. Foamy Trem2^−/−^ cells showed a lipid-loading phenotype by increased expression of *Fabp5*, *Stard4* and *Plin2* but had reduced expression of efflux genes (*Abca1* and *Abcg1*) and upregulation of cholesterol synthesis genes (*Cyp51* and *Hmgcr*) (Fig. 8c). Comparison between WT and Trem2^−/−^ macrophages (untreated or foamy) revealed numerous classical and alternative activation pathways being upregulated in Trem2^−/−^ BV2s, independent of treatment condition (Extended Data Fig. 8a,b). In addition, cell cycle genes (*Ccnd1* and *Ccnd2*) were upregulated in WT BV2 cells. Consistent with the interpretation of dysfunctional cholesterol sensing and response, Trem2^−/−^ BV2s showed upregulation of cholesterol biosynthesis pathways, whereas WT cells showed significant downregulation, as observed by gene set enrichment analysis (GSEA) (Fig. 8d). Interestingly, we also found that Trem2^−/−^ BV2s expressed lower levels of matrix metalloprotease genes (*Mmp9* and *Mmp12*), suggesting a potential role from Trem2 signaling in extracellular matrix remodeling (Extended Data Fig. 8d).

Pathway analysis comparing WT foamy and Trem2^−/−^ foamy macrophages revealed that the top 10 pathways enriched in WT non-foamy and foamy macrophages were associated with cell cycle pathways (Fig. 8e,f and Extended Data Fig. 8c), supporting our previous in vivo conclusions. Finally, we also assessed cytokine production in the supernatant of BV2 cell cultures and found no significant differences between the production of pro-inflammatory and anti-inflammatory cytokines between WT and Trem2^−/−^ BV2s, regardless of lipid loading (Extended Data Fig. 8e). Overall, RNA-seq analysis revealed marked changes in cell proliferation and lipid metabolism pathways in Trem2^−/−^ BV2 cells compared to WT.

**Extended Data Fig. 9:**
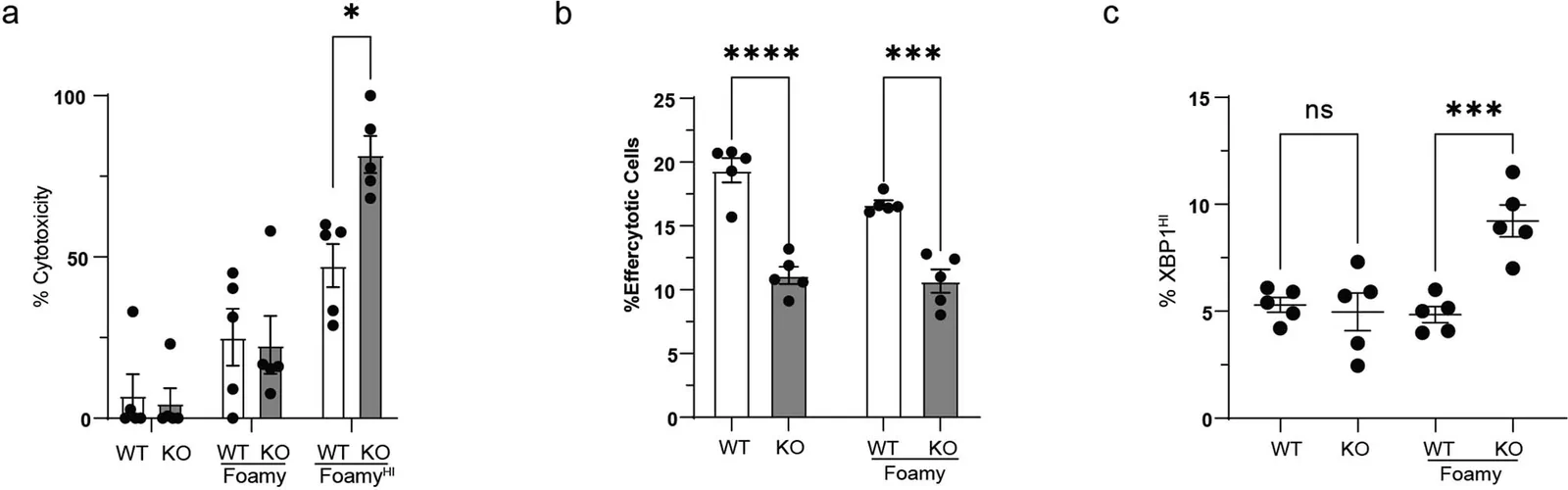
Trem2^-/-^ peritoneal macrophages recapitulate BV2 phenotypes. **a**) WT or Trem2-/- peritoneal macrophages were differentiated in media control, media with 20 µg/mL or 80 µg/mL soluble cholesterol to induce foamy macrophage formation. Cell supernatant was assessed for cytotoxicity by LDH assay after 16 hours (n = 5 biological replicates/group). Data are mean ± S.E.M. Two-tailed ANOVA, P = * < 0.05. **b**) WT or Trem2-/- peritoneal macrophages were differentiated in media control or media with 20 µg/mL soluble cholesterol, then cultured with irradiated, cell trace violet (CTV) labeled splenocytes for 2 hours. Percentage of efferocytotic cells were determined by the % of peritoneal macrophages that were positive for CTV labeled splenocytes (n = 5 biological replicates/group). Data are mean ± S.E.M. Two-tailed ANOVA, P = *** < 0.001, ****<0.0001. **c**) WT or Trem2-/- peritoneal macrophages were differentiated in media control or media with 20 µg/mL soluble cholesterol, then assessed for activation of ER stress response by sXBP1 levels by flow cytometry. Tunicamycin was used as a positive control (n = 5 biological replicates/group). Data are mean ± S.E.M. Two-tailed ANOVA, P = *** < 0.001. Source data

Because gene expression analysis suggested a defect in Trem2^−/−^ BV2 cells in cell cycle regulation and lipid sensing, we first tested whether these cells were more sensitive to lipid loading. Using an LDH assay, we tested cytotoxicity in foamy and non-foamy BV2 knockout and WT cells (Fig. 8g). Soluble cholesterol loading bypasses any defects associated with Trem2^−/−^ lipid loading and forces cell accumulation of cholesterol. Data showed no change in Trem2^−/−^ BV2 cell cytotoxicity until cells were given greater loads of soluble cholesterol (Foamy^Hi^), whereas Trem2^−/−^ BV2 foamy cells showed an approximately 20% increase in overall death in culture. Similar results were observed in peritoneal macrophages lacking Trem2 (Extended Data Fig. 9a). In accordance with this, we also assessed the ability of Trem2^−/−^ and WT foamy or non-foamy BV2 cells to uptake oxLDL and found that only foamy, and not non-foamy, Trem2^−/−^ cells had a defect in DiI-oxLDL uptake (Fig. 8h), consistent with previous peritoneal macrophage data. These observations led us to hypothesize that impaired oxLDL uptake may be linked to reduced survival under lipid-loaded conditions.

Given Trem2’s established role in regulating phagocytosis and efferocytosis^41^, we next asked if Trem2^−/−^ macrophages had a defect in efferocytosis of dead cells by culturing irradiated, cell trace violet (CTV)-stained splenocytes with Trem2^−/−^ or WT BV2s and measuring uptake. We found that, in both non-foamy and foamy state, Trem2 deficiency led to a decreased ability to efferocytose dying cells in BV2s (Fig. 8i) and peritoneal macrophages (Extended Data Fig. 9b), suggesting that, unlike oxLDL uptake, efferocytic impairment may be a product of intrinsic Trem2 deficiency rather than driven by impaired survival.

Because Trem2^−/−^ foamy macrophages showed enhanced cytotoxicity and defective cholesterol response, we hypothesized that loss of Trem2 in foamy macrophages may lead to accumulation of free cholesterol in the ER and promote the ER stress response. This is supported by previous work showing that Trem2-deficient microglia were unable to adapt to excess cholesterol exposure^42^. Cholesterol-mediated cytotoxicity is commonly associated with an ER stress response. We performed intracellular flow cytometry for sXBP1, to indicate ER stress response, in WT and Trem2^−/−^ non-foamy or foamy BV2 cells, and used tunicamycin treatment as a positive control (Fig. 8j)^43^. We observed enhanced ER stress response in Trem2^−/−^ foamy BV2s after 20 µg ml^−1^ soluble cholesterol (Fig. 8j) and in Trem2^−/−^ foamy peritoneal macrophages (Extended Data Fig. 9c). Lipid toxicity can affect a wide variety of organelles, so, to determine if ER stress is the primary mediator of impaired survival seen in lipid-loaded Trem2^−/−^ BV2s, we performed an overnight foamy macrophage formation assay in WT or Trem2^−/−^ cells in the presence or absence of 4-phenylbutyrate (PBA), an ER stress inhibitor^42,44^. PBA treatment led to a minor survival benefit in WT cells, but it recovered Trem2^−/−^ foamy macrophage viability to WT levels in (Fig. 8k), suggesting that ER stress drives the impaired cell survival observed with Trem2 deficiency.

Our sequencing data and other work support that Trem2 signaling drives liver X receptor (LXR) activation and cholesterol efflux^45,46^ and that LXR deficiency can exacerbate ER stress responses^47,48^. Thus, we hypothesized that Trem2 deficiency leads to impaired LXR activation and cholesterol efflux, driving ER stress responses and cell death. GSEA analysis of RNA-seq studies found that Trem2^−/−^ foamy cells failed to induce genes associated with cholesterol efflux (Fig. 8l), LXRα (*Nr1h2*) and LXRβ (*Nr1h3*) (Fig. 8m). To test if driving LXR activation could rescue Trem2^−/−^ foamy macrophages, we cultured WT or Trem2^−/−^ foamy macrophages with the LXR agonist T0901317. LXR agonist treatment led to improved cell survival (Fig. 8n) and decreased ER stress responses by sXBP1 expression in Trem2^−/−^ foamy BV2s (Fig. 8o). Together, these data support a model where Trem2 is required for intracellular lipid sensing and metabolic programming to drive LXR activation in foamy macrophages, which promotes foamy cell survival and maximization of lipid storage, efflux potential and survival.

## Discussion

Macrophages are major contributors to the formation of atherosclerotic plaque. Many features of lipid-loaded foamy macrophage function are well established, including cholesterol uptake, storage and efflux. However, factors specifically regulating foamy macrophage differentiation and survival have remained understudied. We approached this subject using an in silico analysis approach of scRNA-seq data. By generating a meta-scRNA-seq dataset, we were able (1) to achieve finer resolution to identify rare cell clusters, (2) to observe previously concealed intermediate cells and (3) to split previously defined clusters into subclusters. We tested the differentiation trajectory between major atherosclerosis-associated myeloid subsets using computational modeling to predict gene expression trajectories associated with the major inflammatory or foamy macrophage states that appear to be terminal differentiation points.

One substantial result from our analysis was the predicted binary differentiation pathways from recruited monocytes toward inflammatory or foamy macrophage lineages. Studies in other chronic inflammatory disease models, including lung fibrosis, suggest that monocytes undergo a transient inflammatory state before maturing into a pro-resolving macrophage^49^. Additional studies are needed to test this hypothesis by using fate-mapping approaches to determine whether foamy and inflammatory clusters are indeed terminal differentiation states or whether there is plasticity between clusters. Given the highly inflammatory state of cluster 1 defined in Fig. 1c, it may not be surprising if many of these cells undergo inflammasome-mediated pyroptosis^50,51^. Expanded investigation into these possibilities and defining the function of the MHC-II^+^ subsets will be necessary. Pseudotime analysis was used to identify genes associated with commitment toward foamy or inflammatory outcomes. We also performed a genome-wide CRISPR screen to detect genes that regulate the uptake of oxLDL. Together, the screen and pseudotime analysis emphasized the importance of Trem2 in foamy cell formation. Through in vitro and in vivo approaches, we validated the role of Trem2 in regulating foamy macrophage lipid, cellular metabolism and survival in lesions.

Trem2^−/−^ mice possess a variety of phenotypes that make analysis and interpretation difficult, particularly in the context of atherosclerosis^22^. A recent preprint article showed that Trem2^−/−^ was sufficient to drive accelerated necrotic core formation in atherosclerosis^52^. These data further support our findings that Trem2 is required for foamy macrophage survival and efficient efferocytosis. Interestingly, we report that deletion of Trem2 on macrophages does not recapitulate the systemic cholesterol effects seen with whole-body Trem2 deletion^22,52^. Furthermore, our studies did not address advanced lesion formation; thus, results observing no change in necrotic core formation may be expected when compared against the more advanced lesions studied with the Trem2^−/−^ mouse^52^. This leads us to hypothesize that differences between models could be due to developmental defects that require Trem2, such as in brain or liver, or that Trem2 deletion influences cell function in CX3CR1^−^ macrophages, which did not delete in our model. Despite differences between Trem2 models, our findings align and support that Trem2 is a master regulator of lipid-associated macrophage function and phenotype across disease subtypes.

Mechanistically, we found that Trem2 signaling promotes proliferation and survival of foamy macrophages. Deletion of Trem2 led to a defect in LXR-mediated cholesterol efflux and downregulation of cholesterol synthesis pathways. Other groups have also reported deficiencies in cholesterol efflux pathways in Trem2-deficient macrophages^46,53^. Our studies further revealed an increase in ER stress responses in Trem2^−/−^ foamy macrophages, which drove cell death upon lipid loading. Consistent with these results, studies of microglia found that Trem2 deletion leads to impaired uptake and storage of myelin debris^42^. Based on this, we conclude that Trem2 deficiency drives two downstream outcomes that contribute to atherosclerosis protection. Trem2 is required for cholesterol efflux pathway activation, and, in Trem2^−/−^ cells, this impairment leads to exacerbated ER stress response. Cholesterol accumulation in the ER and subsequent ER stress likely promotes the downregulation of cholesterol uptake pathways, such as CD36 (Extended Data Fig. 2h), and contributes to reduced cholesterol uptake. Thus, we propose that changes in lesion size are mediated by slowed cholesterol uptake and enhanced cell death, which are products of reduced cholesterol efflux capacity in Trem2^−/−^ macrophages.

Trem2 has been proposed as a therapeutic target for a variety of disease models, including Alzheimer’s disease^20^ and cancer^54^. Targeting Trem2 is tempting because of its immunomodulatory function. Importantly, both Trem2 agonistic and blocking antibodies have been developed for use in Alzheimer’s disease and cancer. Trem2 blocking antibodies enhance tumor immunotherapy action through modulating the cancer microenvironment^54^. Our results support that targeting Trem2 in established atherosclerotic lesions may result in protection (Fig. 7). However, in advanced lesions, inhibiting Trem2 may lead to enhanced necrotic core formation as a result of impaired cell survival^52^.

In conclusion, loss of Trem2 in foamy macrophages led to enhanced cellular stress response, reduced proliferative potential and augmented cell death. Conditional deletion of Trem2 in foamy macrophages showed attenuated atherosclerosis progression and that targeting Trem2 in established lesions was sufficient to reduce overall plaque burden. Thus, Trem2 is a regulator of foamy macrophage survival and is an appealing target for future therapeutic studies.

## Methods

All experiments and procedures were approved by the University of Minnesota (UMN) Institutional Animal Care and Use Committee and the UMN Institutional Biosafety Committee.

### Animals

Mouse strains used for this study include: B6 (C57BL/6, The Jackson Laboratory (JAX), 000664); Ldlr^−/−^ (B6.129 S7-Ldlr^tm1Her^/J, JAX, 002207); CX3CR1^creER^ (B6.129P2(C)-Cx3cr1^tm2.1(cre/ERT2)Jung^/J, JAX, 020940); Trem2^−/−^ (developed and provided by Marco Colonna, Washington University in St. Louis)^40^; Trem2^flox^ (B6(C3)-Trem2^tm1c(EUCOMM)Wtsi^/AdiujJ, developed and provided by Bruce Lamb at Indiana University, JAX, 029853); R26^tdTomato^ (B6.Cg-Gt(ROSA)26Sor^tm9(CAG-tdTomato)Hze^/J, JAX, 007909); and LysM^cre^ (Lyz2, B6.129P2-Lyz2^tm1(cre)Ifo^/J, JAX, 004781). All mice are on the C57BL/6 background and bred in specific pathogen-free animal facilities maintained by UMN Research Animal Resources. When possible, littermates were used for experiments. Facilities were maintained at ~23 °C with a 12-h light/dark cycle. Cages were changed weekly, and water was freely available through a Lixit valve. Number of animals needed for in vivo assays was estimated using the Vanderbilt power calculator—effect size of 20%, type I error (alpha) of 0.2, internal standard deviation (delta) of 0.15 and a power of 0.9. This analysis suggested the use of a minimum of eight animals per group to detect reasonable differences in our atherosclerosis studies. Experiments were performed in male and female mice in equal numbers.

### HFD and TAM-HFD feeding

In all experiments, animals were fed ad libitum. HFD (diet no. TD.88137; adjusted calories diet, 42% from fat) and TAM-HFD (diet no. TD.130903; adjusted calories diet, 42% fat, tamoxifen-citrate 400 mg kg^−1^) were purchased from Envigo Teklad. Thirty CX3CR1^creER^ Trem2^fl/fl^ Ldlr^−/−^ and 30 littermate controls were enrolled in studies between age 6 weeks and 8 weeks and continuously maintained on HFD through the course of experiments, typically 8 weeks or 16 weeks as described. In conditional Trem2 deletion experiments, age-matched littermate CX3CR1^creER/+^Trem2^fl/fl^Ldlr^−/−^ mice were used for the experimental group (Trem2^ΔMac^), whereas CX3CR1^creER/+^Trem2^fl/+^Ldlr^−/−^ or CX3CR1^+/+^Trem2^f/f^Ldlr^−/−^ mice were combined for the Cntl group.

### scRNA-seq data integration and analysis

Raw files were downloaded from the National Center for Biotechnology Informationʼs Sequence Read Archive. The kallisto bustools (version 0.46.1) workflow was used for the quantification of each sample in each dataset. The count matrices obtained from the kallisto bustools pipeline were used as input. For the preparation of the atherosclerotic meta-dataset and samples integration, the Seurat package (version 3.1.5) was used. Samples from each study were processed and integrated into study-related objects, which are available in Single Cell Navigator (https://artyomovlab.wustl.edu/scn/).

#### Seurat analysis

Droplets with ambient RNA and noisy cells were filtered using the EmptyDrops function from the DropletUtils R package, and then genes that expressed in fewer than 200 cells were removed. The fraction of mitochondrial genes was calculated for every cell, and cells with a mitochondrial fraction that was more than the sample-specific threshold defined by the confidence interval were filtered out. All samples were normalized using the SCTransform function. We next processed the data, and features were detected using SelectIntegrationFeatures. A list with all samples as its elements was prepared for integration using the PrepSCTIntegration and FindIntegrationAnchors functions. Finally, samples were integrated using the IntegrateData function. Principal component analysis (PCA) was run on the integrated object. For two-dimensional visualization of object structure, both t-distributed stochastic neighbor embedding (tSNE) and uniform manifold approximation and projection (UMAP) approaches were implemented using the first 20 principal components. For clustering purposes, the functions FindNeighbors and FindClusters were used.

#### Identification of plaque monocytes and myeloid macrophages

All clusters were manually annotated using canonical gene markers. T cells (*Cd3d*^+^), B cells (*Cd79a*^+^), smooth muscle cells (*Sparc*^+^), proliferating cells (*Mki67*^+^), monocytes (*Treml4*^+^, *Ly6c2*^+^ and *Sell*^+^) and different macrophages subtypes (*Retnla*^+^, *Adgre1*^+^, *Lyve1*^+^ and *Fabp5*^+^) were identified in the prepared meta-dataset. We first identified and removed the barcodes from T cells, B cells, smooth muscle cells as well as proliferating cells. We then used the expression of markers (*Lyve1*^+^ and *Mrc1*^*high*^) to separate adventitia macrophages from intima macrophages. The remaining barcodes (assumed to be monocytes and intima macrophages) were later re-analyzed from the very beginning (using the same steps as outlined above). Populations were found to be monocytes, intima macrophages and DCs. Cells that correspond to monocyte/macrophage populations were extracted and fully re-analyzed using Seurat (using the same steps outlined above). For the final iteration, clusters were manually annotated using expression levels of known myeloid markers of monocytes, foamy macrophages and inflammatory macrophages.

#### Trajectory analysis

All cells from the object that contained monocytes and intima macrophages were used, and the infer_trajectory function from the dyno package (version 0.1.2) was used on the normalized counts (integrated assay, data slot) with the available slingshot singularity container (version 1.0.3). Trajectory visualization was implemented after dimensionality reduction by UMAP using the dimred_umap function. We also used the dyno package to calculate gene importance scores for foamy or inflammatory differentiation along pseudotime^30^. In brief, the scores are calculated using a random forest regression model trained on gene expression values to predict pseudotime values.

#### Differential expression across pseudotime

To find genes that are differentially expressed across pseudotime, the TradeSeq package (version 1.4.0) was used. Raw counts (RNA assay, counts slot) were used as input expression, and the design matrix corresponding to the study was used as fixed effects to remove the batch effect. We used earlyDETest to identify genes that are differentially expressed early after the branch point.

#### Human scRNA-seq analysis

We took the counts data for symptomatic and asymptomatic human atherosclerotic carotid endarterectomy samples from Gene Expression Omnibus GSE224273 (ref. ^32^). To identify the samples from the study (and which samples are symptomatic and asymptomatic), we compared the barcodes with the file ‘merged_plaque_gex-umi-data-mnn-cat_macrophages.txt’ from the supplement published in figshare. To filter out dying cells and cells of lower quality, we used the usual filtering procedure based on the number of genes detected per cell and mitochondrial (mt) content; all samples had <15% mt content. Filtering boundaries were selected for each sample manually.

To adjust for donor effect, we used integration methods with SCTransform normalization. In brief, all six objects were normalized using scTransform; 3,000 integration features were selected using SelectIntegrationFeatures; PrepSCTIntegration was applied to the object list; anchors were found using FindIntegrationAnchors; and then data were integrated using IntegrateData. We then followed the usual workflow of PCA, UMAP for the first 30 components of PCA and FindNeighbors for the first 30 components of PCA, followed by FindClusters with resolution of 0.6. We ended up with 20 clusters, four of which (namely, 2, 8, 11 and 13) were identified as myeloid cells based on the expression of *CD14* and *MRC1*. We then checked expression levels of *FABP5*, *LGALS3* and *TREM2* and identified cluster 8 as foamy macrophages. Because differential expression tests in scRNA-seq are biased and prone to report many false positives^55^, we decided to use the pseudobulk approach to test differential expression using DESeq2. First, we split the cells from cluster 8 based on their samples. Then, counts for each cell from the same samples were added together, resulting in six pseudobulk samples. Next, we performed differential expression analysis for RNA-seq using DESeq2. We used the data from ‘merged_plaque_gex-umi-data-mnn-cat_macrophages.txt’ to say which patients were symptomatic. The DESeq2 differential expression test was run using ‘symptomatic’ as the only variable, and lfcShrink was used with type = ‘normal’.

### Bone marrow chimera

Ldlr^−/−^ mice (recipients) were lethally irradiated with 1,100 rad using an X-ray irradiator using a split dose (550 rad each) delivered 5 h apart. Mice were rested for 4 h and then adoptively transferred with donor bone marrow. Donor bone marrow cells (5 × 10^6^) were i.v. injected in a 100-µl volume to irradiated recipient mice. Mice were allowed to reconstitute for 8 weeks and then transitioned to HFD for atherosclerosis studies.

### Serum cytokine and cholesterol analysis

Blood was rested to clot at room temperature for 1 h, and then samples were centrifuged at 1,000*g* for 10 min at 4 °C in a tabletop centrifuge (Beckman Coulter). The supernatant (serum) was collected and assessed for cytokines and cholesterol content. Total cholesterol analysis was performed using the Wako/Fujifilm Cholesterol-E kit (999-02601), following the manufacturer’s instructions. Multiplex cytokine analysis was performed using LEGENDplex murine inflammatory panel (BioLegend, 740446), following the manufacturer’s protocol and analysis pipeline.

### In vitro culture

BV2 cells were a kind gift from Herbert Virgin (Washington University in St. Louis). BV2 cells were cultured in non-treated 10-cm tissue culture plates (VWR, 10062-880) in 5% FBS (Corning, sourced from the United States with low endotoxin) in DMEM (Sigma-Aldrich, D0819), with addition of 1% penicillin–streptomycin (Sigma-Aldrich, P4333), HEPES (Sigma-Aldrich, H0887) and MEM non-essential amino acids (Sigma-Aldrich, M7145). Peritoneal cells were collected by lavage with HBSS with 2% FBS and 2 mM EDTA as previously described and grown overnight in the same media as BV2 cells for assays. For foamy cell formation assay, cells were plated at 1 × 10^6^ cells in a 24-well plate (VWR, 10861-558) and treated overnight with 20 µg ml^−1^ soluble cholesterol. In brief, methyl-beta-cyclodextrin was incubated with cholesterol at a 1:6 ratio and then stored at −20 °C. DiI-labeled oxLDL (Kalen Biomedical, medium oxidized, 770282-9) was added to BV2 or peritoneal macrophages at 10 µg ml^−1^ for 4 h. Cells were recovered from plates with a 3-min incubation with 0.25% trypsin EDTA (Sigma-Aldrich, T4045) and then scraped with rubber policeman to lift cells, washed with media and stained for analysis.

### Generation of cas9 BV2 cell line

BV2 cells were transduced by lentivirus with Cas9 (pLX_311cas9) and selected with blasticidin (2 µg ml^−1^) for 10 d. A single clone was isolated by dilution cloning. Cas9 expression was validated by flow cytometry using intracellular staining of cas9 protein with anti-cas9 monoclonal antibody (7A9-3A3, Cell Signaling Technology, 48796). Cas9 activity was assessed by transducing the cas9-BV2 cells with pXPR_047, which expresses eGFP, and a small guide RNA (sgRNA) targeting eGFP. After transduction, cells were selected with puromycin (2 µg ml^−1^) for 8 d. The percentage of GFP^+^ cells was assessed by flow cytometry on an LSR II/Fortessa.

### Library preparation

The mouse Gouda (CP0074 33) genome-wide CRISPR knockout library containing two sgRNAs per gene (purchased from the Broad Institute) was lentivirally transduced into 9 × 10^7^ cas9-BV2 cells at a low multiplicity of infection (MOI), resulting in approximately 30% of the cells infected and achieving 500-fold coverage after puromycin selection. At 24 h after transduction, infected cells were selected with puromycin (2 µg ml^−1^) for 5 d.

### CRISPR screen

BV2 cells containing the Gouda library were treated with 20 µg ml^−1^ soluble cholesterol overnight to generate foamy macrophages. Cells were then pulsed for 4 h with DiI-oxLDL (Kalen Biomedical). Cells were then sorted for oxLDL uptake, as indicated by DiI labeling. The high and low 9% of DiI-labeled cells were selected and then lysed for guide sequencing. Genomic DNA (gDNA) was purified, and guides were sequenced with directed primers at the Broad Institute. Data were analyzed using the Model-based Analysis of Genome-wide CRISPR–Cas9 Knockout (MAGeCK) algorithm^56^. Raw read counts were first median normalized to harmonize sample variations regarding library size and count distribution. Next, a negative binomial approach involving mean-variance modeling was applied to determine the sgRNA abundance difference between the control group and the test group. Subsequently, statistical scores were calculated and used to rank sgRNAs using the MAGeCK test. We chose positively ranked genes as they represent the test (DiI^Low^) group. Log fold change values reported by MAGeCK are used to perform pathway analysis using the MAGeCK pathway function.

### Generation of Trem2^−/−^ BV2 cell line

Targeted deletion strategy was adapted from previous work^57^. Cas9-BV2 cells were transfected with sgRNA targeting Trem2 (sgRNA sequence: CGTGTGTGCTCACCACACGC). To prepare the transfection, sgRNA was placed into the RNA backbone (pRDA_118) by mixing 8 µg of guide RNA backbone with 2 µl of BsmBI enzyme and 5 µl of NE Buffer 3.1 in a total volume of 50 µl in water and incubated at 55 °C overnight. After incubation, 1 µl (10,000 U ml^−1^) of CIP was added and incubated for 1 h at 37 °C. Digested sample was run on a 1% agarose gel and selected for ~8-kilobase size. The band was cut from the gel and purified using a gel extraction kit (Qiagen, 28704). DNA was annealed and phosphorylated using forward and reverse oligos (CACCGCGTGTGTGCTCACCACACGC and AAACGCGTGTGGTGAGCACACACGC) in a ramp PCR setup increasing temperature by 0.1 °C per second. The sample was then ligated with sgRNA overnight using T4 ligase. Stbl3 *Escherichia coli* was transformed by adding 5 µl of ligation reaction mix to 50 µl of *E. coli* cells. Cells were rested on ice for 30 min, and then cells were heat shocked at 42 °C for 30 s and returned to ice. Cells were transferred to growth media and left in a shaking incubator for 1 h at 37 °C. Next, 100 µl of cells was spread on an LB agar plate with 200 µg ml^−1^ ampicillin for selection. Guide RNA clones were selected after 24 h and expanded in culture. Plasmids were isolated (Qiagen Miniprep Kit, 27106) and sequenced for appropriate insertion. Zymo PurePlasmid Miniprep Kit (D4209) was used to isolate endotoxin-fee plasmids for clones that were carried forward in the assay.

Cas9-BV2 cells were plated at 3 × 10^5^ in a six-well plate in DMEM (without penicillin–streptomycin) + 2 µg ml^−1^ blasticidin. Transfection was performed using warm TransIT-LTI (Mirus Bio, MIR2304) and then vortexed. Next, 5 µg of plasmid DNA was placed in 250 µl of OptiMEM media (Gibco, 31985062), and then 7.5 µl of transit-LTI reagent was added to DNA/OptiMEM solution. Samples were incubated at room temperature for 20 min and then given to cas9-BV2 cells. Cells were incubated for 3 d. Puromycin was added to treatment wells and incubated for an additional 5 d for selection in appropriate insertion. Deletion efficiency for Trem2-targeting sgRNA was validated by flow cytometry and TIDE analysis using Sanger sequencing of the pooled clones, verifying ~60% deletion efficiency. Individual Trem2^−/−^ clones were isolated by limiting dilution and expansion of single clone wells. Deletion of Trem2 was validated by flow cytometry.

### Bulk RNA-seq collection and analysis

BV2 cells were lysed for RNA isolated by TRIzol directly from plates after overnight treatment in media or cholesterol. Samples were submitted to the UMN Genomics Core for RNA isolation (Qiagen RNeasy Kit) and sequencing using the NovaSeq platform. Raw data were processed using the CHURP pipeline developed by the Minnesota Supercomputing Institute, which implemented and integrated Trimmomatic, HISAT2, SAMTools and featureCounts. Data were aligned to the *Mus musculus* GRCm38 (Ensembl release 102) mouse reference genome. Differential expression analysis was adopted from DEseq2 (version 1.32.0). Pathway analyses were performed using the fgsea package (version 1.18.0).

### Flow cytometry

Single-cell suspensions were filtered through 100-µm nylon mesh (McMaster-Carr) and then washed in FACS buffer (HBSS with 2% FBS and 2 mM EDTA). Supernatant was discarded, and cell pellets were stained for 30 min at 4 °C, protected from light. Antibodies were stained at 1 mg ml^−1^ in a volume of 50 µl, unless a specified concentration was specified by the manufacturer. Conventional flow cytometry was performed on a BD LSRFortessa or a BD LSRFortessa X-20. Spectral cytometry was collected using a Cytek Aurora. All machines are supported and maintained by the UMN Flow Cytometry Core facility. Data were processed in FlowJo (Tree Star) or Cytek SpectroFlo software. The following antibodies were used: Trem2 APC rat anti-mouse (clone 237920, R&D Systems); Trem2 FITC rat anti-mouse (clone 78.18, eBioscience); CD68 rat anti-mouse (clone FA-11, BioLegend); Ki67 rabbit anti-mouse (clone SP6, Abcam); CD45 BV480 rat anti-mouse (clone 30-F11, BioLegend); CD11b BV605 rat anti-mouse (clone M1/70, BioLegend); Ly6G BV785 rat anti-mouse (clone 1A8, BioLegend); Ly6C BV421 rat anti-mouse (clone HK1.4, BioLegend); CD115 PerCPCy5.5 rat anti-mouse (clone AFS98, BioLegend); TCRβ APC hamster anti-mouse (clone H57-597, BioLegend); CD19 FITC rat anti-mouse (clone 1D3, BioLegend); and sXBP1 AF647 rat anti-mouse (clone E9V3E, Cell Signaling Technology).

### Cytotoxicity assay

Cell viability was assessed using an LDH cytotoxicity detection kit (Sigma-Aldrich) per the manufacturerʼs protocol. In brief, WT or Trem2^−/−^ BV2 cells were plated in 96-well plates and cultured overnight either in media alone or in media with 20 µg ml^−1^ or 40 µg ml^−1^ cholesterol. The next day, supernatants were transferred to a new 96-well plate, along with wells of lysed cells and media alone for positive and negative controls. Samples were then treated with 100 µl of the reaction mixture and incubated at room temperature for 20 min. Then, samples were treated with 50 µl of stop solution, and absorbance was immediately recorded on a SpectraMax Plus plate reader at 492 nm. Percent cytotoxicity was determined using the absorbance values minus the background controls and normalized to baseline per the manufacturer’s instructions (% cytotoxicity = (sample value − negative control) / (positive control − negative control) × 100). For ER stress and cytotoxicity studies with phenylbutyrate (Sigma-Aldrich), cells were cultured overnight in 10 μM PBA or vehicle before analysis. For LXR agonist studies, cells were cultured overnight with the LXR agonist T0901317 (Sigma-Aldrich) at 10 μM.

### Efferocytosis assay

Efferocytosis was measured by seeding 2 × 10^5^ BV2 cells or peritoneal macrophages in 24-well plates and co-culturing them with 1 × 10^6^ irradiated, CTV-labeled B6 splenocytes for 2 h at 37 °C. After co-culture, cells were washed three times and then stained with Live/Dead ghost dye and F4/80 for analysis via flow cytometry for determination of macrophages that have taken up CTV^+^ splenocytes.

### Aortic sinus imaging/immunofluorescence

To examine plaque formation at the aortic sinus, hearts from atherosclerotic mice were either fixed overnight in 4% paraformaldehyde (PFA)/30% sucrose solution in 1× PBS or directly embedded. Hearts were embedded in O.C.T. and frozen and then sectioned on a cryostat at 10-µm thickness. For staining, slides were warmed to room temperature for 10 min on the benchtop. Samples were briefly fixed in 4% PFA for 2 min and then washed with 1× PBS. Samples were blocked with 5% donkey serum and permeabilized with 1% Triton X-100 for 30 min. Samples were washed two times with 1× PBS. Primary antibodies were diluted 1:500 in PBS, and the samples were stained for 1 h. Samples were washed three times with 1× PBS and then stained with secondary antibodies conjugated to fluorochromes (1:1,000 dilution) for 30 min. Samples were washed three times and then mounted with Fluoromount (Southern Biotech) and imaged. Samples were imaged using a Leica SP8 inverted confocal microscope (fluorescence imaging) or with an attached bright-field light source and camera. Plaque area was measured using the ImageJ analysis tool, where researchers were blinded to the samples and identified areas of interest manually. For plaque analysis, the two-maximum cross-section lesion area for each sample was averaged and used for data presentation.

### Aorta plaque analysis

Aortas were surgically removed from mice and fixed in 4% PFA overnight at 4 °C. Periadipose tissue was removed manually under a dissecting microscope (Leica S9i). Samples were then cut open and pinned en face to wax dishes. Samples were washed three times with water and then incubated in propylene glycol for 5 min. Next, the aortas were submerged in Oil Red O (Sigma-Aldrich, O1516) for 3 h and protected from light. Afterwards, the dishes were washed in 85% propylene glycol for 5 min, followed by three washes with water. Images were taken using a Leica S9i stereo microscope with a 10-megapixel color camera. Images were merged using Adobe Photoshop and analyzed using ImageJ software. Plaque area was quantified by laboratory staff members who were blinded to the sample identify.

### Human atherosclerotic plaque samples

Human plaque samples were isolated from the cranial circle of Willis and donated for research as part of the UMN anatomy bequest program. These tissues were collected postmortem and de-identified with limited patient history. Sample displayed was from a 93-year-old female with known history of cardiovascular disease. Samples were fixed in 4% formalin, dissected to approximately 1-mm size and then stained for CD68 and Trem2 and imaged by confocal microscopy in whole mount. Images were analyzed using Bitplane Imaris version 9 software. Human carotid atherosclerotic plaques were collected during thromboendarterectomy procedures at the University Hospital of Nice after approval from the local ethics committees and in agreement with the Declaration of Helsinki. Informed written consent was obtained from all patients. Patients had either symptomatic carotid stenosis or very tight asymptomatic stenosis. Carotid atherosclerotic plaques were immediately snap frozen in liquid nitrogen. Samples were cross-sectioned at 8-µm thickness using a Leica 3050S cryostat. For DAB staining, sections were incubated for 10 min in PBS containing 0.3% H_2_O_2_ and rinsed in buffer for 5 min. Sections were then incubated in 2.5% normal horse serum for 10 min and rinsed again in buffer for 5 min. The sections were then incubated in primary antibody buffer containing 1.5% normal horse serum and anti-human Trem2 antibody or isotype control at 10 µg ml^−1^ overnight at 4 °C. Then, biotinylated pan-specific universal secondary antibody was added for 20 min. After washing, sections were incubated with streptavidin/peroxidase complex for 5 min, and then peroxidase substrate was applied for 4 min, followed by counterstaining with hematoxylin.

### Statistics and reproducibility

Graphs were generated and statistical analysis performed in GraphPad Prism software. In general, comparison between two experimental groups used a Student’s *t*-test, whereas comparisons of more than two groups used a two-tailed ANOVA analysis. Graph error bars represent s.e.m., and *P* values were considered statistically significant below 0.05. In graphs, *P < 0.05, **P < 0.01, ***P < 0.001 and ****P < 0.0001. Each experiment was repeated three independent times, unless specified otherwise.

### Reporting summary

Further information on research design is available in the Nature Portfolio Reporting Summary linked to this article.

## Supplementary information

Reporting Summary

Supplementary Table 1 CRISPR screen guide enrichment.

## Source data

Source Data Fig. 2 Statistical source data.

Source Data Fig. 3 Statistical source data.

Source Data Fig. 4 Statistical source data.

Source Data Fig. 5 Statistical source data.

Source Data Fig. 6 Statistical source data.

Source Data Fig. 7 Statistical source data.

Source Data Fig. 8 Statistical source data.

Source Data Extended Data Fig./Table 4 Statistical source data.

Source Data Extended Data Fig./Table 6 Statistical source data.

Source Data Extended Data Fig./Table 7 Statistical source data.

Source Data Extended Data Fig./Table 8 Statistical source data.

Source Data Extended Data Fig./Table 9 Statistical source data.

## Data Availability

Newly generated gene expression data (bulk RNA-seq) have been made available in the Gene Expression Omnibus repository (GSE231659). All other data supporting the findings in this study are included in Source Data.
