# Supplementary material for: Trem2 promotes foamy macrophage lipid uptake and survival in atherosclerosis
Source: Nat Cardiovasc Res. 2023 Oct 30;2(11):1015–31. doi: 10.1038/s44161-023-00354-3 (PMC11031198; doi:10.1038/s44161-023-00354-3)
Supplement: Supplementary file 1 [file 44161_2023_354_MOESM1_ESM.pdf]

nature portfolio

Corresponding author(s): Jesse W. Williams

Last updated by author(s): 09/20/2023

Reporting Summary

Nature Portfolio wishes to improve the reproducibility of the work that we publish. This form provides structure for consistency and transparency in reporting. For further information on Nature Portfolio policies, see our Editorial Policies and the Editorial Policy Checklist.

Statistics

For all statistical analyses, confirm that the following items are present in the figure legend, table legend, main text, or Methods section.

n/a	Confirmed	
☐	☒	The exact sample size (*n*) for each experimental group/condition, given as a discrete number and unit of measurement
☐	☒	A statement on whether measurements were taken from distinct samples or whether the same sample was measured repeatedly
☐	☒	The statistical test(s) used AND whether they are one- or two-sided<br>*Only common tests should be described solely by name; describe more complex techniques in the Methods section.*
☐	☒	A description of all covariates tested
☐	☒	A description of any assumptions or corrections, such as tests of normality and adjustment for multiple comparisons
☐	☒	A full description of the statistical parameters including central tendency (e.g. means) or other basic estimates (e.g. regression coefficient) AND variation (e.g. standard deviation) or associated estimates of uncertainty (e.g. confidence intervals)
☐	☒	For null hypothesis testing, the test statistic (e.g. *F*, *t*, *r*) with confidence intervals, effect sizes, degrees of freedom and *P* value noted<br>*Give P values as exact values whenever suitable.*
☒	☐	For Bayesian analysis, information on the choice of priors and Markov chain Monte Carlo settings
☒	☐	For hierarchical and complex designs, identification of the appropriate level for tests and full reporting of outcomes
☒	☐	Estimates of effect sizes (e.g. Cohen's *d*, Pearson's *r*), indicating how they were calculated

Our web collection on statistics for biologists contains articles on many of the points above.

Software and code

Policy information about availability of computer code

Data collection	Software used for data collection are described in the methods of the manuscript. For flow cytometry, BD Bioscience FACS DIVA (v8), for imaging Leica LAS X.
Data analysis	Data analysis software are outlined and described in the manuscript. For flow cytometry FlowJo (treestar) v10.6.2, for image processing Leica LAS X and Imaris (bitplane) v9, graphs and statistics were run using Graphpad Prism v8, Image quantification performed with ImageJ, scRNAseq and RNAseq data were processed using Cell Ranger and Seurat Packages.

For manuscripts utilizing custom algorithms or software that are central to the research but not yet described in published literature, software must be made available to editors and reviewers. We strongly encourage code deposition in a community repository (e.g. GitHub). See the Nature Portfolio guidelines for submitting code & software for further information.

Data

Policy information about availability of data

All manuscripts must include a data availability statement. This statement should provide the following information, where applicable:

- Accession codes, unique identifiers, or web links for publicly available datasets
- A description of any restrictions on data availability
- For clinical datasets or third party data, please ensure that the statement adheres to our policy

All scRNA-seq datasets have been uploaded and made publicly available for download in the Gene Expression Omnibus (GEO) repository with accession numbers included in the methods section of the manuscript. Bulk RNA-seq data has been submitted to GEO and made available, with accession number in the manuscript.

Human research participants

Policy information about studies involving human research participants and Sex and Gender in Research.

Reporting on sex and gender	*Use the terms sex (biological attribute) and gender (shaped by social and cultural circumstances) carefully in order to avoid confusing both terms. Indicate if findings apply to only one sex or gender; describe whether sex and gender were considered in study design whether sex and/or gender was determined based on self-reporting or assigned and methods used. Provide in the source data disaggregated sex and gender data where this information has been collected, and consent has been obtained for sharing of individual-level data; provide overall numbers in this Reporting Summary. Please state if this information has not been collected. Report sex- and gender-based analyses where performed, justify reasons for lack of sex- and gender-based analysis.*
Population characteristics	*Describe the covariate-relevant population characteristics of the human research participants (e.g. age, genotypic information, past and current diagnosis and treatment categories). If you filled out the behavioural & social sciences study design questions and have nothing to add here, write "See above."*
Recruitment	*Describe how participants were recruited. Outline any potential self-selection bias or other biases that may be present and how these are likely to impact results.*
Ethics oversight	*Identify the organization(s) that approved the study protocol.*

Note that full information on the approval of the study protocol must also be provided in the manuscript.

Field-specific reporting

Please select the one below that is the best fit for your research. If you are not sure, read the appropriate sections before making your selection.

☒ Life sciences ☐ Behavioural & social sciences ☐ Ecological, evolutionary & environmental sciences

For a reference copy of the document with all sections, see nature.com/documents/nr-reporting-summary-flat.pdf

Life sciences study design

All studies must disclose on these points even when the disclosure is negative.

Sample size	For in vivo atherosclerosis experiments power calculations were performed to determine the number of animals needed for assays were estimated using the Vanderbilt power calculator; effect size of 20%, type I error (alpha) of 0.2, internal standard deviation (delta) of 0.15, and a power of 0.9. RNA-seq was performed in triplicate or quadruplet, based on prior experience with needed replicates to assess genetic changes. Other in vitro experiments were performed in quadruplet (or more), which was sufficient to observe significant differences between tested groups.
Data exclusions	No data/samples are excluded from any experiments presented in the manuscript.
Replication	Independent experiment replication was performed for all experiments (2 or more independent times). All data is provided as a concatinated dataset when possible. Otherwise, data derived from a representative experiment is shown. Replication of independent experiments were typically performed two or three independent times, with 3 or more animals for all replicates. Details with exact numbers will be included in the figure legend of each experiment.
Randomization	For animal experiments, mice of different genotypes were cohoused during all experiments. All experiments were associated with genetic models of deletion, thus no randomization was utilized in organizing the in vivo experiments.
Blinding	Researchers analyzing atherosclerotic plaque area in the aortic root and aortic arch were blinded to the sample ID. Each in vivo experiment was independently analyzed by two researchers in the lab. For quantification of foamy macrophage bone marrow chimera, samples were initially scored for foamy macrophage identity, then followed by determination of tdTomato positive or negative.

Reporting for specific materials, systems and methods

We require information from authors about some types of materials, experimental systems and methods used in many studies. Here, indicate whether each material, system or method listed is relevant to your study. If you are not sure if a list item applies to your research, read the appropriate section before selecting a response.

Materials & experimental systems

n/a	Involved in the study
☐	☒ Antibodies
☐	☒ Eukaryotic cell lines
☒	☐ Palaeontology and archaeology
☐	☒ Animals and other organisms
☒	☐ Clinical data
☒	☐ Dual use research of concern

Methods

n/a	Involved in the study
☒	☐ ChIP-seq
☐	☒ Flow cytometry
☒	☐ MRI-based neuroimaging

Antibodies

Antibodies used	Antibody info is provided in the experimental methods.
Validation	Antibodies were provided by manufacturer with quality control data, which can be access from the supplier.

Eukaryotic cell lines

Policy information about cell lines and Sex and Gender in Research

Cell line source(s)	BV2 cell line was used for CRISPR screen and in vitro assays. Provided by Dr. Herbert Virgin (Washington University).
Authentication	Cells were validated to express BV2-specified genes, including Trem2 at baseline.
Mycoplasma contamination	Cells were not assessed for mycoplasma
Commonly misidentified lines (See ICLAC register)	Unknown

Animals and other research organisms

Policy information about studies involving animals; ARRIVE guidelines recommended for reporting animal research, and Sex and Gender in Research

Laboratory animals	Male and female mice were used for this study and all animals were enrolled in experiments between 6-10 weeks of age. Animals were housed in Specific Pathogen Free (SPF) facility with ambient temperature ~23-24-degrees Celsius, with 12-hour light / 12-hour dark cycle, food available ad libitum, humidity not reported. Cages were changed weekly and water was freely available through Lixit valves. Animal care and oversight was performed by the University of Minnesota Research Animals Resources (RAR).<br><br>Mouse strains used for this study include; B6 (C57BL/6, Jackson Laboratory (Jax) 000664), Ldlr-/- (B6.129S7-Ldlrtm1Her/J, Jax 002207), CX3CR1creER (B6.129P2(C)-Cx3cr1tm2.1(cre/ERT2)Jung/J, Jax 020940), Trem2-/- (developed and provided by Dr. Colonna, Washington University)31, Trem2flox (B6(C3)-Trem2tm1c(EUCOMM)Wtsi/AdiujJ, developed and provided by Dr. Lamb at Indiana University, Jax 029853)17, R26tdTomato (B6.Cg-Gt(ROSA)26Sortm9(CAG-tdTomato)Hze/J, Jax 007909), LysMcre (Lyz2, B6.129P2-Lyz2tm1(cre)Ifo/J, Jax 004781).
Wild animals	N/A
Reporting on sex	Sex was addressed in the in vivo experiments of the study, with no significant changes observed between male and female. Thus, primary figures provided include combined data between sexes. Supplemental data provide some sex-segregated data.
Field-collected samples	N/A
Ethics oversight	Animal use was approved by the University of Minnesota Medical School Institutional Animal Care and Use Committee.

Note that full information on the approval of the study protocol must also be provided in the manuscript.

Flow Cytometry

Plots

Confirm that:

☒ The axis labels state the marker and fluorochrome used (e.g. CD4-FITC).

☒ The axis scales are clearly visible. Include numbers along axes only for bottom left plot of group (a 'group' is an analysis of identical markers).

☒ All plots are contour plots with outliers or pseudocolor plots.

☒ A numerical value for number of cells or percentage (with statistics) is provided.

Methodology

Sample preparation	Whole blood was collected from the facial vein into EDTA treated tubes and 10 µL were used for cell count using a fluorescent Nexcelom cellometer. Red blood cells (rbc) were lysed using 1x RBC lysis buffer (Biolegend) for 4 minutes on the benchtop. For aorta digestions, following dissection, samples were cut into small pieces and incubated at 37 °C for 4 min with 45 gentle shaking in enzyme cocktail (containing DNase I (90 U/mL), collagenase I (675 U/mL), collagenase XI (187.5 U/mL), hyaluronidase (90 U/mL)). Following digestion samples were filtered through 100 micron mesh to remove dead cells and debris. Prior to staining, all single cell suspensions were blocked with anti-CD16/32 antibody (2.4G2, Biolegend) diluted 1:1,000, then stained for specific antigens. Antibodies were used at 1 µg/mL in 50 µL volume, and stained for 30 minutes on ice. Samples were washed and analyzed by flow cytometry. The following antibodies were used: Trem2 APC rat anti-mouse (clone 237920, R&D systems CAT#FAB17291A), Trem2 FITC rat anti-mouse (clone 78.18, eBioscience,CAT#MA528223), CD68 rat anti-mouse (clone FA-11, Biolegend,CAT#137001), Ki67 rabbit anti-mouse (clone SP6, Abcam,CAT#ab16667), CD45 BV480 rat anti-mouse (clone 30-F11, Biolegend,CAT#566168), CD11b BV605 rat anti-mouse (clone M1/70, Biolegend,CAT#101237), Ly6G BV785 rat anti-mouse (clone 1A8, Biolegend,CAT#127645), Ly6C BV421 rat anti-mouse (clone HK1.4, Biolegend,CAT#128031), CD115 PerCPCy5.5 rat anti-mouse (clone AFS98, Biolegend,CAT#135525), TCRβ APC hamster anti-mouse (clone H57-597, Biolegend,CAT#109211), CD19 FITC rat anti-mouse (clone 1D3, Biolegend,CAT#152403), sXBP1 AF647 rat anti-mouse (clone E9V3E, Cell Signaling,CAT#38139S). Specificity for Trem2 antibody was validated against Trem2-/- mice and cell lines.
Instrument	BD LSR Fortessa and BD LSR X-20 were used for data collection.
Software	Data collected with BD FACSDiva and analyzed using FlowJo.
Cell population abundance	N/A
Gating strategy	Blood and aorta samples were gated by FSC-A/SSC-A to select cells based on general characteristics. Doublets were removed by gating against FSC-W/SSC-H and FSC-H/SSC-W. Then samples were selected for populations of interest based on fluorescent antibody labeling, typically CD45+. Further strategy is provided in the figures and supplement of the manuscript.

☒ Tick this box to confirm that a figure exemplifying the gating strategy is provided in the Supplementary Information.
